# miR‐484 in Hippocampal Astrocytes of Aged and Young Rats Targets CSF‐1 to Regulate Neural Progenitor/Stem Cell Proliferation and Differentiation Into Neurons

**DOI:** 10.1111/cns.70415

**Published:** 2025-04-30

**Authors:** Jiahua Qu, Zhichao Lu, Yongbo Cheng, Song Deng, Wei Shi, Qianqian Liu, Yuejuan Ling

**Affiliations:** ^1^ Research Center of Clinical Medicine, Co‐Innovation Department of Neurosurgery Affiliated Hospital of Nantong University, Medical School of Nantong University Nantong China; ^2^ Institute of Pain Medicine and Special Environmental Medicine Nantong University Nantong China

**Keywords:** aging, astrocytes, CSF‐1, miR‐484, neural progenitor/stem cells

## Abstract

**Aim:**

Aging‐related cognitive decline is closely linked to the reduced function of neural progenitor/stem cells (NPSCs), which can be influenced by the neural microenvironment, particularly astrocytes. The aim of this study was to explore how astrocytes affect NPSCs and cognitive function during aging.

**Methods:**

H_2_O_2_‐treated astrocytes were used to mimic the aging phenotype of astrocytes. Proteomic analysis identified altered protein expression, revealing high levels of colony‐stimulating factor‐1 (CSF‐1) in the supernatant of H_2_O_2_‐treated astrocytes. Primary NPSCs were isolated and cultured in vitro, then stimulated with varying concentrations of recombinant CSF‐1 protein to assess its effects on NPSC proliferation, differentiation, and apoptosis. Transcriptome sequencing identified miR‐484 related to CSF‐1 in H_2_O_2_‐treated astrocytes, and a dual‐luciferase assay verified the interaction between miR‐484 and CSF‐1. The impact of miR‐484 overexpression on NPSC function and cognitive restoration was evaluated both in vitro and in vivo (in 20‐month‐old rats).

**Results:**

High concentration of CSF‐1 inhibited the NPSC proliferation and differentiation into neurons while inducing apoptosis. Overexpression of miR‐484 downregulated CSF‐1 expression by binding to its 3' untranslated region, thereby promoting the NPSC proliferation and differentiation into neurons. In 20‐month‐old rats, miR‐484 overexpression improved spatial learning and memory in the Morris water maze, increased NPSC proliferation, and reduced apoptosis.

**Conclusion:**

Our findings reveal that miR‐484 regulates CSF‐1 to influence NPSC proliferation, differentiation into neurons, and apoptosis, consequently improving cognitive function in 20‐month‐old rats. This study provides a foundation for developing therapeutic strategies targeting age‐related hippocampal cognitive impairments.

## Graphical abstract

miR‐484 was shown to negatively regulate CSF‐1 expression and promote NPSC neurogenesis, decreasing NPSC apoptosis.

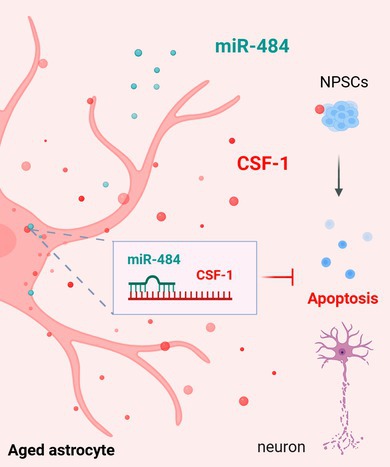

## 1 Introduction

With the increasing average lifespan and the growing proportion of the older adult population, health issues stemming from age‐related brain dysfunction are on increase [1, 2, 3]. Neurogenesis in adults predominantly occurs in the subventricular zone adjacent to the lateral ventricle and in the dentate gyrus of the hippocampus. However, as individuals age, this process steadily diminishes [4], largely because neural stem cells (NSCs) lose their ability to proliferate [5]. This reduction in neurogenesis is thought to play a significant role in the onset of age‐related cognitive deficits and memory deterioration [6].

The neural microenvironment within the central nervous system (CNS) significantly influences the proliferation and differentiation of NSCs [7]. Astrocytes, as a major component of this neural microenvironment, are adept at sensing changes and play a critical role in maintaining CNS homeostasis [8]. However, the functionality of astrocytes declines with age, suggesting that age‐related dysfunction in these cells may contribute to the decline in NSC function [9]. Therefore, understanding the impact of astrocytes on the decreased proliferation and differentiation of NSCs is crucial for addressing the cognitive deficits associated with hippocampal function.

Notably, studies have consistently shown that colony‐stimulating factor‐1 (CSF‐1) is a key factor in regulating normal growth and brain development [10, 11, 12]; however, under pathological conditions, astrocytes regulate the production of CSF‐1, which contributes to the progression of experimental autoimmune encephalomyelitis [13]. Available evidence indicates that the modulation of immunity by CSF‐1 may have neuroprotective effects on Parkinson's disease [14]. Nevertheless, Alzheimer's disease, a neurodegenerative disorder, is associated with increased CSF‐1 expression. This upregulation may exacerbate neuronal damage by promoting the activation of glial cells and inflammatory responses [15]. These research findings reveal that CSF‐1 acts as both a guardian and a disruptor in the nervous system: it can exert regulatory functions and neuroprotection, but it may also induce pathogenic effects. More importantly, recent research has shown that in nerve injury, astrocytic CSF‐1 expression can be downregulated by miR‐214‐3p, which attenuates neuroinflammation and pain [16]. Additionally, miR‐122 is downregulated in Alzheimer's disease and is notably correlated with specific inflammatory molecules (GM‐CSF, INF‐α2, IL‐1α, IL‐8, and MIP‐1β) [17]. However, the direct regulatory role of miRNAs on CSF‐1 expression in the context of age‐related neurodegenerative diseases remains to be elucidated.

**FIGURE 1:**
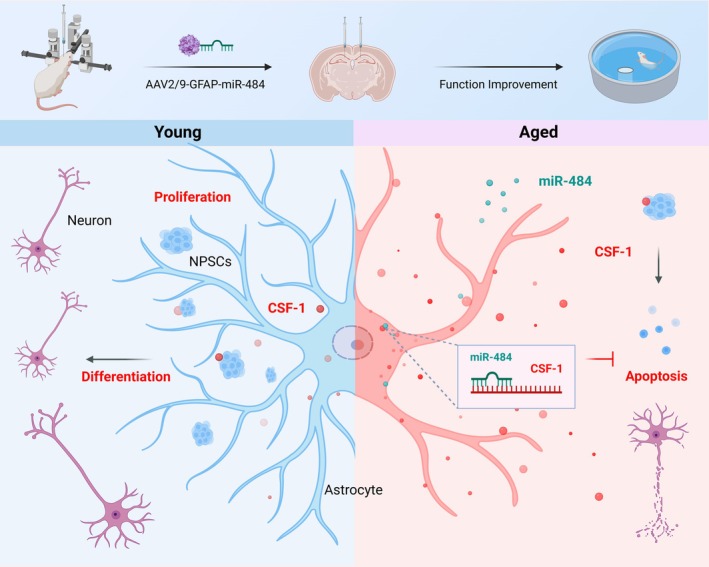
miR‐484 regulated NPSC neurogenesis by targeting CSF‐1. Injecting AAV2/9‐GFAP‐miR‐484 into the hippocampi of aged rats resulted in improved cognitive function. The appropriate levels of CSF‐1 in young astrocytes promoted the NPSC proliferation and differentiation into neurons. Conversely, high levels of CSF‐1 in aged astrocytes induced apoptosis in NPSCs. miR‐484 overexpression inhibited the CSF‐1 secretion from astrocytes in aged rats, increased NPSC neurogenesis, decreased NPSC apoptosis, and enhanced learning and memory abilities in aged rats (Figure was created with BioRender.com).

In this study, we treated astrocytes with H_2_O_2_ to mimic the aging phenotype of astrocytes, as H_2_O_2_ is a commonly used agent for inducing cellular senescence [18]. We found that H_2_O_2_‐treated astrocytes induced the neural progenitor/stem cells (NPSCs) senescence and apoptosis. Subsequently, we further identified the significantly expressed protein CSF‐1 in the supernatant of H_2_O_2_‐treated astrocytes through proteomic analysis. Furthermore, we confirmed the effects of CSF‐1 on NPSC proliferation, differentiation into neurons, and apoptosis. Through RNA sequencing and dual‐luciferase reporter assays, we have identified miR‐484 as an upstream regulator that can specifically target and bind to CSF‐1. Our study shows that miR‐484 regulates CSF‐1, affecting NPSC proliferation, differentiation into neurons, and apoptosis, which enhances cognitive function in 20‐month‐old rats. These findings suggest novel molecular targets and promising research directions for promoting the functional recovery of NPSCs (Figure 1).

## 2 Materials and Methods

### 2.1 Experimental Animals

The experiments were conducted using Sprague–Dawley (SD) rats at different stages: embryonic day 12 (E12, *n* = 10), E17 (*n* = 10), 1‐day‐old neonatal (*n* = 20), 4‐week‐old male (*n* = 40) and 20‐month‐old male (*n* = 40). The animals were supplied by the Nantong University Animal Experiment Center. NPSCs were isolated from E12 embryos, primary neurons from E17 embryos, and primary astrocytes and microglia from 1‐day‐old neonatal rats. In vivo experiments, including behavioral tests and adeno‐associated virus injection, were performed in 4‐week‐old and 20‐month‐old rats. The animals were kept in cages equipped with filters and automatic lighting, following a 12 h light–dark cycle. The Ethics Committee of Laboratory Animals of Nantong University approved all experimental procedures (Approval No. S20240116‐012).

### 2.2 Culture of Hippocampal NPSCs

E12 rats (*n* = 10) were used for the extraction of NPSCs [19]. The hippocampal tissue was separated, dissociated into a single‐cell suspension, and centrifuged at 1200 r/min for 3 min. Subsequently, the supernatant was discarded, and the cells were resuspended in NPSC culture medium (DMEM/F12 1:1, 2% B27, 20 μg/L basic fibroblast growth factor‐2, 20 μg/L epidermal growth factor) before being grown in T25 culture flasks. The culture medium was replaced with fresh NPSC culture medium every 3 days. Neurospheres were gathered following a week of in vitro cultivation.

### 2.3 Culture of Primary Astrocytes, Primary Microglia, and Primary Neurons

We extracted primary astrocytes from the brains of 1‐day‐old neonatal rats (*n* = 20). The primary astrocyte preparation has been previously detailed [20]. Briefly, the cerebral cortex tissue underwent digestion using 0.25% trypsin at 37°C for 10 min, after which it was filtered through a 70 μm cell strainer. Then, the isolated cells were seeded in 25 cm^2^ culture flasks coated with 0.01% poly‐L‐lysine and cultured in DMEM/F12 medium under culture conditions of 37°C and 5% CO_2_. By the third passage, the cells are ready for experimentation. We assessed the purity of primary astrocytes using GFAP staining (Figure S3B).

The primary microglia preparation has been previously detailed [21]. We used 1‐day‐old neonatal rats to extract primary microglia. Briefly, we carefully extracted the brain and placed the Petri dish containing the brain under a microscope. We then meticulously removed the meninges, collected the cortices, and diced the tissue into small fragments. Following a 20 min digestion period with 0.25% trypsin at 37°C, the cells were seeded into 25 cm^2^ flasks that had been pre‐coated with 0.01% poly‐L‐lysine. The cells were cultured in DMEM/F12 for a duration of 2 weeks, with the medium being changed every 2–3 days. Upon reaching confluence, the flasks were subjected to shaking at 37°C and 180 rpm for 30 min to separate microglia. We assessed the purity of primary microglia using IBA1 staining (Figure S3A).

The preparation method for primary neurons was slightly different [22]. Primary neurons were prepared from the hippocampus of E17 rats. Briefly, the isolated hippocampus was finely diced in dissociation medium and subsequently treated with a solution of papain (41 U/mL) and L‐cysteine 0.06% (p/v) for 3 min at 37°C. Next, the samples underwent three washes, were then incubated for 4 min with slow shaking, and mechanically dissociated. Cells were plated onto dishes that had been pre‐coated with 0.01% poly‐L‐lysine and cultured in DMEM/F12 supplemented with glutamine (2 mM), sodium pyruvate (1%), glucose (20 mM), and inactivated horse serum (10%). After 4 h, the medium was replaced by Neurobasal medium containing B27 (2%), glutamine (2 mM) and penicillin and streptomycin. The cultures were utilized for experiments between 7 and 10 days post‐seeding. We assessed the purity of primary neurons using Tuj1 staining (Figure S3C).

### 2.4 Culture of H_2_O_2_ ‐Treated Astrocytes, H_2_O_2_ ‐Treated Microglia, and H_2_O_2_ ‐Treated Neurons

We treated primary cells with H_2_O_2_ to mimic the aging phenotype, as H_2_O_2_ is a commonly used agent for inducing cellular senescence [18]. Primary astrocytes, microglia, and neurons induced to senescence with 3% H_2_O_2_ treatment are referred to as H_2_O_2_‐treated astrocytes, H_2_O_2_‐treated microglia, and H_2_O_2_‐treated neurons, respectively. The specific experimental procedures involved stimulating primary cells with complete medium containing 200 μM of 3% H_2_O_2_ for 4 h, followed by washing the cells three times with PBS. Cell culture medium without H_2_O_2_ was then added for 24 h of cultivation to obtain senescent cells.

### 2.5 Collection of Supernatants From Primary and H_2_O_2_ ‐Treated Astrocytes

After the primary astrocytes had grown to around 80% confluence at the third passage, the culture medium was changed to fresh DMEM/F12 without serum. After 24 h, the supernatant of primary astrocytes was collected. While collecting the H_2_O_2_‐treated astrocytes, their supernatant was also collected. The collected supernatant was treated by spinning cell debris down with centrifugation at 2000g for 10 min and stored at −80°C. The collection of supernatants from primary and H_2_O_2_‐treated astrocytes was used for proteomic analysis and qRT‐PCR.

### 2.6 H_2_O_2_ ‐Treated Cells and NPSCs Co‐Cultures

Corning 24‐well plate Transwell co‐culture chambers (0.4 mm) were used for in vitro co‐cultures. NPSCs were placed in the lower chamber (24‐well plate), while H_2_O_2_‐treated astrocytes, microglia, and neurons were placed in the upper chamber. The H_2_O_2_‐treated cells were co‐cultured with NPSCs for 48 h, after which the NPSCs in the lower chamber (24‐well plate) were fixed by 4% paraformaldehyde (PFA) for immunofluorescence staining. We performed Nestin and Ki67 double staining to assess the proliferation ability of NPSCs.

### 2.7 Immunofluorescence Analysis

Rats were anesthetized and subjected to pericardial perfusion with a solution containing 0.9% saline followed by 4% PFA. Following extraction, the brains were postfixed overnight at room temperature in the same fixative. They were then sequentially immersed in a 20% sucrose solution for 2–3 days and subsequently in a 30% sucrose solution for an additional 3–4 days. Each brain was embedded in OCT compound. The tissues were then sectioned into 10 μm slices using a cryostat and stored at −20°C until further use. Cells were fixed by 4% PFA for 10 min. The cells or brain sections were blocked for 2 h with 5% goat serum. The samples were initially incubated overnight at 4°C with primary antibodies. Following this, a 2 h incubation at 37°C in darkness was required for the application of secondary antibodies. DAPI was added and allowed to incubate for 10 min. A fluorescence microscope (DM 5000B; Leica) was used for observation. Table S1 provides a list of the antibodies used in the immunofluorescence analysis.

### 2.8 Drug Administration

The CSF‐1 recombinant protein (Table S1) was dissolved in PBS, and concentrations of 0, 0.05, 0.5, and 5 μg/mL CSF‐1 were added to the NPSC culture medium for 24 h, respectively. We isolated and cultured primary NPSCs in vitro by adding different concentrations of CSF‐1 recombinant protein to determine its effects on NPSCs.

### 2.9 Enzyme‐Linked Immunosorbent Assay (ELISA)

A CSF‐1 ELISA kit (MULTI SCIENCES) was used according to the manufacturer's instructions (Table S1) to detect the expression of CSF‐1 in the supernatant of astrocytes after transfection with a miR‐484 mimic and inhibitor and assess the expression of CSF‐1 in the hippocampal homogenate supernatants of both 4‐week‐old and 20‐month‐old rats.

### 2.10 Quantitative Real‐Time Polymerase Chain Reaction (qRT‐PCR)

Total RNA from cells and tissues was extracted using TRIzol (Vazyme). miRNAs were reverse transcribed using the miRNA 1st Strand cDNA Synthesis Kit (by stem‐loop) (Vazyme) and miRNA RT primer. mRNAs were transcribed into cDNA with the HiScript II Reverse Kit (Vazyme) following the instructions provided by the manufacturer's instructions. We normalized the relative expression of mRNA and miRNA to GAPDH and U6, respectively. The primers synthesized by Sangon Biotech were listed in Table S2.

### 2.11 Western Blotting

Rat hippocampal tissue was extracted and incubated for 30 min on ice using RIPA lysis buffer (Beyotime). Protein concentrations were determined using the bicinchoninic acid protein assay kit (Beyotime). Proteins were separated using SDS‐PAGE and subsequently transferred onto a PVDF membrane. After blocking, the membrane was exposed to the primary antibody (Table S1) overnight at 4°C, followed by incubation with the secondary antibody (Table S1) for 1 h. Protein bands were analyzed using ImageJ software.

### 2.12 β‐Gal Staining

Frozen brain sections were stained using a β‐gal staining kit (Beyotime). The sections were fixed and stained with the β‐gal staining solution for 18 h at 37°C (without CO_2_), according to the kit instructions, followed by blocking the enzyme reaction with PBS on ice. Frozen section images were obtained using microscopy to assess β‐gal activity (the percentage of β‐gal positive cell among all cells) in the hippocampus. We used β‐gal staining to detect the senescence of NPSCs in the hippocampus of 4‐week‐old and 20‐month‐old rats.

### 2.13 Flow Cytometry

We used flow cytometry to detect the apoptosis and proliferation of NPSCs. For analyzing the cell cycle, cells were stained using Propidium Iodide/RNase Staining Buffer (BD Biosciences) following the provided instructions. To analyze apoptosis, cells were stained using a PE Annexin V Apoptosis Detection Kit (BD Biosciences). All stained cells were assessed using the FACSCalibur instrument (BD Biosciences).

### 2.14 Target miRNA Prediction

TargetScan (http://www.targetscan.org) and MirDateBase (http://www.mirdb.org) were utilized for miRNA prediction. The differentially expressed miRNAs in primary and H_2_O_2_‐treated astrocytes were cross‐referenced with miRNAs targeting CSF‐1 predicted by these online databases to identify the potential upstream miRNAs.

### 2.15 miRNA Transfection

We used miRNA transfection to examine the effect of miR‐484 on the function of NPSCs. miR‐484 mimic and miR‐484 inhibitor were purchased from Ribobio (Guangzhou, China). H_2_O_2_‐treated astrocytes were transfected with Lipofectamine 3000 (Invitrogen) reagent according to the manufacturer's guidelines.

### 2.16 Dual‐Luciferase Reporter Assay

The dual luciferase assay was used to verify the interaction between miR‐484 and CSF‐1. The miRNA eukaryotic plasmid and overexpression vector were constructed by Genechem. 293 T cells were categorized into the LUC‐NC group, LUC‐WT group, and LUC‐MUT group. Each group was transfected with plasmids, and the luciferase assay was performed 48 h after cell transfection. The fluorescence values of firefly luciferase and renilla luciferase were detected using a multifunctional microplate reader.

### 2.17 Adeno‐Associated Virus Injection

Rno‐mir‐484 was obtained from the cDNA library of Genechem (Shanghai, China) with the following primers: rno‐mir‐484 forward: 5′‐TCCGCTGCTCGCCGGGATCCGCTCCTCCCATCCCCTTTCTA‐3′ and reverse: 5′‐GAGCAGCGCTCGGTATCGATAAGCTCCGTTCGACCTGGTGTC‐3′. The AAV vector plasmid CV256(pAAV‐GFAP‐polyA) (purchased from Shanghai Genechem Co. Ltd.), the vector, and rno‐mir‐484 gene sequence were digested by BamHI and ClaI restriction enzymes, and complete cloning was performed through the In‐fusion recombination method. The recombinant vector was detected by DNA sequencing. 20‐month‐old rats were randomly divided into two groups: the vector group (*n* = 12) and the overexpression (OE) miR484 group (*n* = 12). Rats were anesthetized with isoflurane gas before being positioned in a stereotaxic device. The incision site was carefully trimmed. The meninges were then treated with 3% H_2_O_2_ to expose the fontanelle, which was used as a landmark for orientation. Virus was injected bilaterally into the DG of the dorsal hippocampi using the following coordinates [23, 24]: (from fontanelle) posterior = −2 mm, lateral = ±1.50 mm (from skull surface) height = −3.5 mm. The virus was administered at a rate of 0.2 μL/min (2 μL/well) using a Hamilton syringe. After the injection was administered, the syringe needle was retained in position for an additional 10 min to facilitate uniform dispersion of the viral solution.

### 2.18 Behavioral Tests

We conducted the shuttle‐box experiment and Morris water maze (MWM) test to assess the rats’ learning and memory capabilities. Rats were randomly divided into four groups: 4‐week group (*n* = 6), 20‐month group (*n* = 6), Vector group (*n* = 6), OE‐miR484 group (*n* = 6). In the shuttle‐box experiment, rats were placed in the dark avoidance shuttle box. After a 10 s interval, the middle door between the light and dark chambers was opened. Upon entering the dark chamber inside, the rats received an electric shock for 2 s. After the electrical stimulation training, the rats were returned to the box with the middle door open, but no shocks were delivered. During a 5 min observation period, we recorded the number of shuttle movements.

The MWM test comprised two phases: training and testing. During training, the platform was placed in the middle of the circular pool's third quadrant. Rats were gently placed into the pool from each of the four quadrants, facing the pool's wall. Rats underwent daily training for four consecutive days, and the latency of escape to the platform was recorded. On the fifth day, during the testing phase, the platform was removed, and each rat was gently placed into the pool at the location where the platform had been. The Animal behavior video analysis system (version ZH‐SBS.V2.0, Anhui Yaokun Biotechnology) was used to track and record the rats’ swimming path for 2 min.

### 2.19 Mass Spectrometry Data Acquisition and Analysis

We performed mass spectrometry analysis between the supernatants of primary and H_2_O_2_‐treated astrocytes. Proteins were in‐gel digested with porcine trypsin. Then purified peptides were transferred to sample vials and analyzed using timsTOF Pro (Bruker, Bremen, Germany). Serum pools were depleted of the most abundant proteins using Agilent Mouse 3 Multiple Affinity Removal System Column following the manufacturer's protocol (Agilent Technologies) [25]. The MS/MS data obtained were processed using the MaxQuant search engine (v1.6.15.0) and were explored against the *Rattus norvegicus* database (uniport database, 29,940 sequence. Download 20201214). Proteins for which fold change > 2 or < 0.5 and *p*‐values < 0.05 were considered to be a differentially expressed proteins.

### 2.20 miRNA Sequencing

RNA sequencing and quality control of the sequences from both primary and H_2_O_2_‐treated astrocytes (*n* = 3 per group) were performed using the Illumina Novaseq 6000 platform. We employed Diana miRpath for the analysis of differentially expressed miRNAs, with a significance threshold of *p*‐values < 0.05. Gene annotation of the sequencing data was conducted using the KEGG database. OE Biotech Co. Ltd. (Shanghai, China) performed sequencing and analysis of small RNA.

### 2.21 Statistical Analysis

Data analysis was performed using GraphPad Prism 9.1 software, with experimental data reported as mean ± standard deviation. To evaluate the normality of continuous variable distributions, the Shapiro–Wilk test was applied. All data were tested for normality; any data that did not exhibit a normal (Gaussian) distribution were analyzed using nonparametric equivalents. *p*‐values < 0.05 were considered statistically significant. Each experiment was conducted at least three times. For data comparisons, unpaired Student's *t*‐tests, one‐way ANOVA, and two‐way ANOVA were utilized.

## 3 Results

### 3.1 The Decline of Learning and Memory Ability in 20‐Month‐Old Rats Is Related to the Decrease of Neurogenesis

**FIGURE 2:**
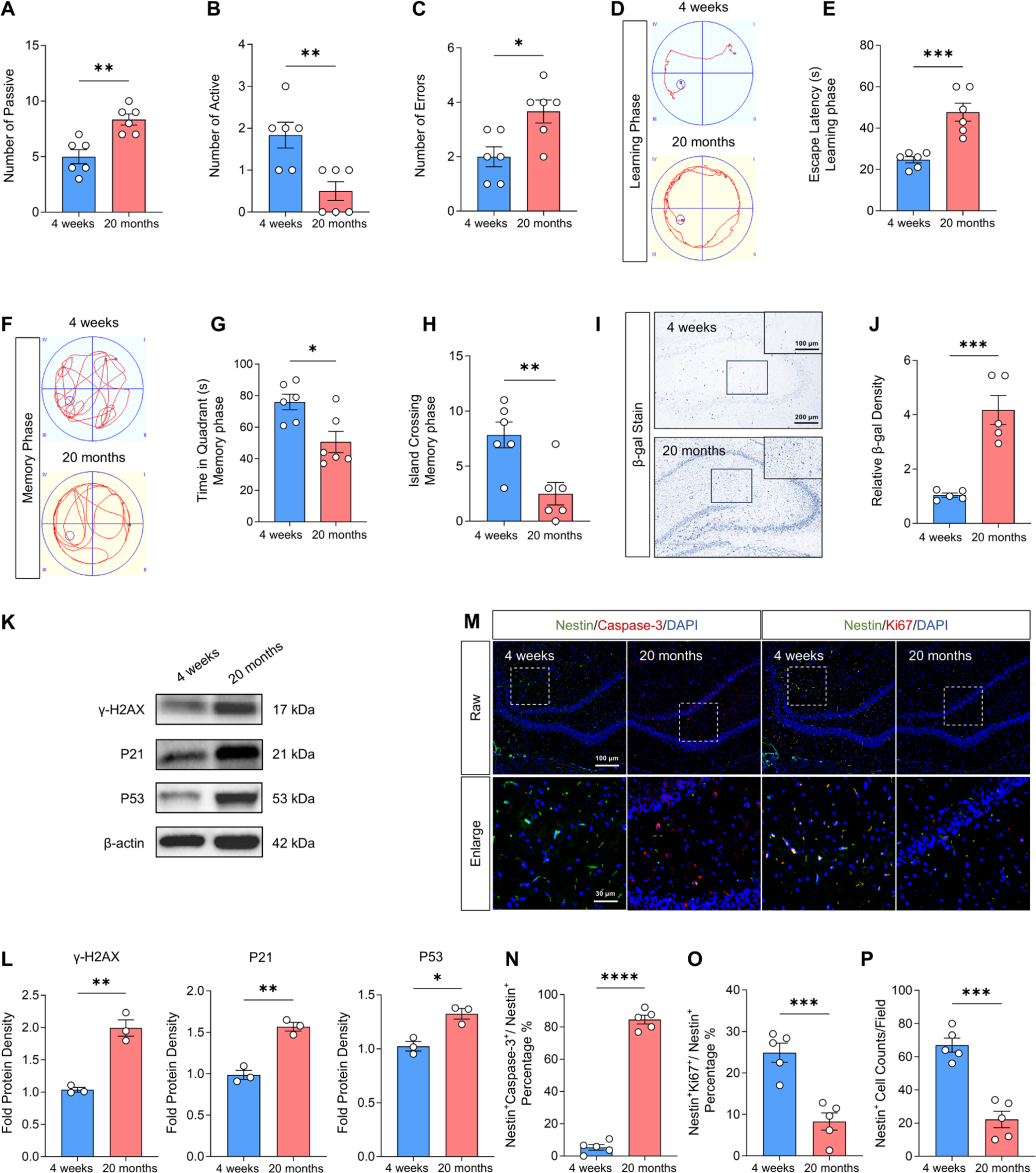
Senescent phenotype and proliferation and apoptosis of hippocampal NPSCs in 4‐week old and 20‐month‐old rats. (A–C) Quantitative statistics of the number of passive, active, and errors in rats. *p < 0.05, **p < 0.01, 20 months versus 4 weeks. Student's *t*‐test (*n* = 6). (D) Representative results of the Morris water maze test during the learning period in rats; (E) Quantitative statistics of escape latency in rats. ***p < 0.001, 20 months versus 4 weeks. Student's *t*‐test (*n* = 6). (F) Representative results of the Morris water maze test during the memory period in rats. (G‐H) Quantitative statistics of dwell time in the target quadrant and number of crossing island in rats. *p < 0.05, **p < 0.01, 20 months versus 4 weeks. Student's *t*‐test (*n* = 6). (I) β‐gal staining of the hippocampus in 20‐month‐old and 4‐week‐old rats. (J) Quantitative statistics of relative β‐gal density. ***p < 0.001, 20 months versus 4 weeks. Student's *t*‐test (*n* = 5). (K) Western blot detection of γ‐H2AX, P21, and P53 in isolated hippocampal neurospheres in the hippocampus of 20‐month‐old and 4‐week‐old rats. (L) Quantitative analysis of the Western blot results. *p < 0.05, **p < 0.01, 20 months versus 4 weeks. Student's *t*‐test (*n* = 3). (M) Immunofluorescence images of Nestin^+^/Caspase‐3^+^ double‐stained cells and Nestin^+^/Ki67^+^ double‐stained cells in the hippocampus. (N–P) Quantitative analysis of the percentage of Nestin^+^/Caspase‐3^+^ double‐stained cells, Nestin^+^/Ki67^+^ double‐stained cells in whole cells and the number of Nestin^+^ cells in each field in the hippocampus. ***p < 0.001, ****p < 0.0001, 20 months versus 4 weeks. Student's *t*‐test (*n* = 5).

In this study, we assessed the senescence of NPSC with aging by using SD rats. In order to understand cognitive functions, we first evaluated the memory capacities of SD rats across various age groups (4 weeks; 20 months) using the shuttle‐box experiment (Figure 2A–C). The results showed that compared to the 4‐week group, the number of active responses decreased, and the number of errors increased in the 20‐month group. We further evaluated spatial learning (Figure 2D) and memory (Figure 2F) in 4‐week old and 20‐month‐old rats using the MWM. As revealed in Figure 2E, the escape latency increased in the 20‐month group compared to that in the 4‐week group during the learning phase. Additionally, during the memory phase, the 20‐month group exhibited a decrease in dwell time in the target quadrant (Figure 2G) and a reduced number of island crossings (Figure 2H) compared with the 4‐week group. These results demonstrated an age‐related decline in cognitive function in rats.

As aging progresses, NPSCs undergo senescence, impairing their intrinsic functions. To investigate age‐related effects on NPSCs, we detected the senescence status of NPSCs in both 4‐week and 20‐month groups through β‐gal staining, immunofluorescence staining, and Western blot. Our results showed that the hippocampus exhibited a higher activity of age‐related β‐gal in the 20‐month group compared to the 4‐week group (Figure 2I,J) and the number of Nestin^+^/β‐gal^+^ double‐stained cells in the hippocampus was significantly increased in the 20‐month group (Figure S1). Furthermore, the expression levels of aging‐related proteins (γ‐H2AX, P21, and P53) [26, 27, 28, 29] in hippocampal cells from the 20‐month group were increased compared to those in the 4‐week group (Figure 2K,L). These results collectively suggest that NPSCs exhibit increased aging in 20‐month‐old rats compared to that in 4‐week‐old rats.

Given that NPSCs contribute significantly to cognition maintenance and restoration, their depletion directly results in cognitive function decline [30, 31]. Therefore, we speculated that cognitive impairment in 20‐month‐old rats is related to the depletion of NPSCs. To investigate the differences in NPSC activity in the hippocampus between 4‐week‐old and 20‐month‐old rats, we examined proliferating and apoptotic NPSCs in the hippocampus of both age groups using immunofluorescence staining. The results revealed that, compared to the 4‐week group, the number of Caspase‐3^+^/Nestin^+^ cells was significantly increased, while the numbers of Ki67^+^/Nestin^+^ cells and Nestin^+^ cells were greatly reduced in the 20‐month‐old group (Figure 2M–P). These results cumulatively indicate age‐related NPSC senescence in rats and suggest that senescence may lead to decreased proliferation ability and increased apoptosis of NPSCs, ultimately resulting in cognitive dysfunction.

### 3.2 Astrocytes Contribute to Senescence and Apoptosis of NPSCs in the Hippocampal Microenvironment of 20‐Month‐Old Rats

**FIGURE 3:**
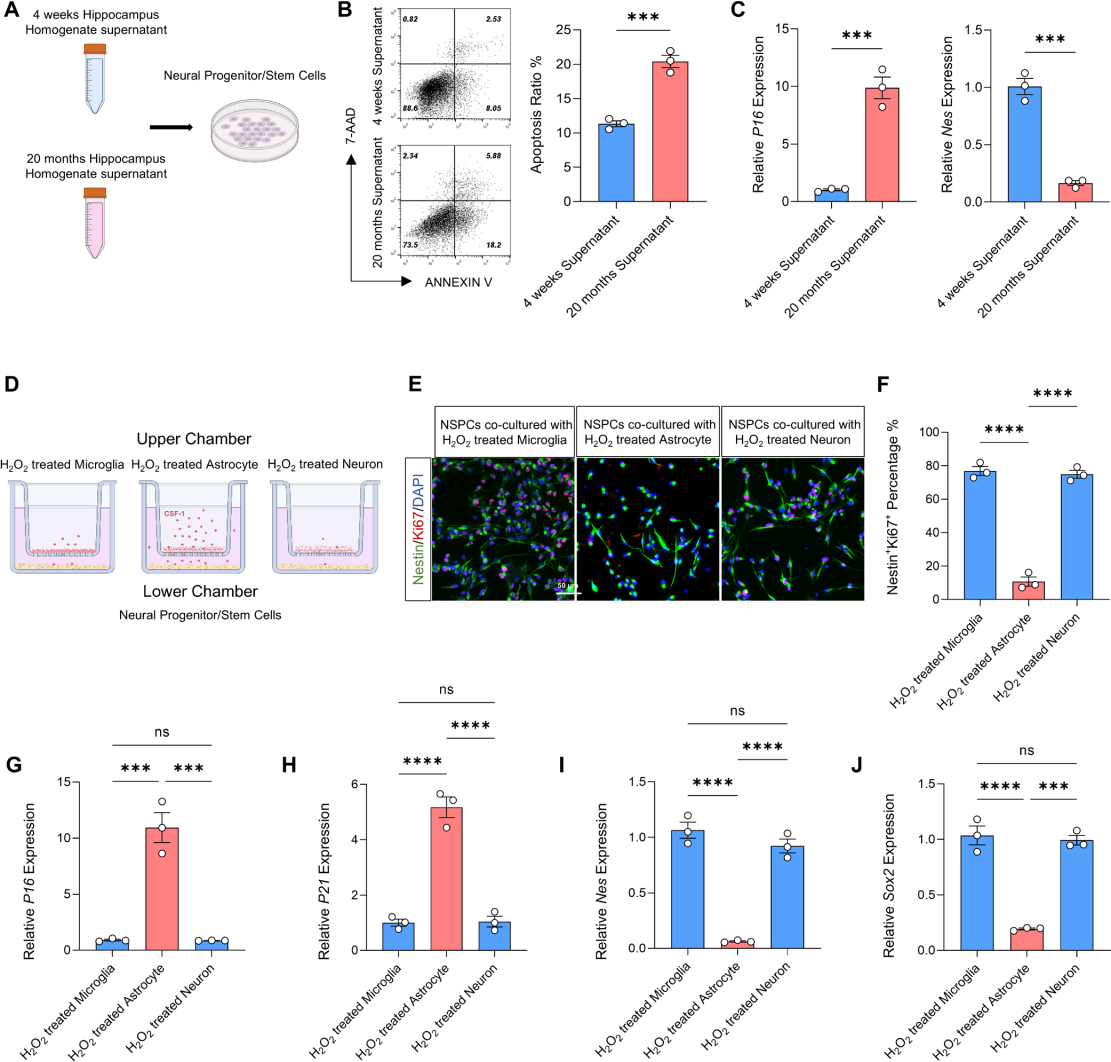
Effects of the hippocampal microenvironment of 20‐month‐old and 4‐week‐old rats on senescence and apoptosis of NPSCs. (A) Diagram of hippocampal homogenate supernatants from 20‐month‐old and 4‐week‐old rats co‐cultured with NPSCs. (B) The apoptosis of NPSCs co‐cultured with 4‐week supernatant and NPSCs co‐cultured with 20‐month supernatant was analyzed and quantified by flow cytometry. ***p < 0.001, 20‐month supernatant versus young supernatant. Student's *t*‐test (*n* = 3). (C) qRT‐PCR detection of the expression of p16 and Nes in NPSCs co‐cultured with 4‐week supernatant and in NPSCs co‐cultured with 20‐month supernatant. ***p < 0.001, 20‐month supernatant versus 4‐week supernatant. Student's *t*‐test (*n* = 3). (D) Diagram of H_2_O_2_‐treated microglia, H_2_O_2_‐treated astrocytes, and H_2_O_2_‐treated neurons co‐cultured with NPSCs. (E) Immunofluorescence images of Nestin^+^/Ki67^+^ double‐stained cells of NPSCs co‐cultured with H_2_O_2_‐treated microglia, H_2_O_2_‐treated astrocytes, and H_2_O_2_‐treated neurons. (F) Quantitative analysis of the percentage of Nestin^+^/Ki67^+^ double‐stained cells in whole cells. ****p < 0.0001, H_2_O_2_‐treated astrocytes versus H_2_O_2_‐treated microglia, H_2_O_2_‐treated neurons versus H_2_O_2_‐treated microglia, H_2_O_2_‐treated astrocytes versus H_2_O_2_‐treated neurons. One‐way ANOVA followed by Bonferroni's tests (*n* = 3). (G–J) qRT‐PCR detection of the expression of p16, p21, Nes, and Sox2 in NPSCs co‐cultured with H_2_O_2_‐treated microglia, H_2_O_2_‐treated astrocytes and H_2_O_2_‐treated neurons. ***p < 0.001, ****p < 0.0001, H_2_O_2_‐treated astrocytes versus H_2_O_2_‐treated microglia, H_2_O_2_‐treated neurons versus H_2_O_2_‐treated microglia, H_2_O_2_‐treated astrocytes versus H_2_O_2_ treated neurons. One‐way ANOVA followed by Bonferroni's tests (*n* = 3).

With aging, the hippocampal microenvironment varies significantly between physiological periods, and the hippocampal microenvironment significantly influences the activity of NPSCs. To investigate the impacts of the hippocampal microenvironment on the apoptosis of NPSCs in 4‐week‐old and 20‐month‐old rats, we co‐cultured primary NPSCs with hippocampal homogenate supernatants from both 4‐week‐old and 20‐month‐old rats. The apoptosis and senescence of NPSCs were assessed using flow cytometry and qRT‐PCR. Our results indicated that compared to NPSCs co‐cultured with the hippocampal homogenate supernatants of 4‐week‐old rats, apoptosis and senescence were increased in NPSCs co‐cultured with the hippocampal homogenate supernatants of 20‐month‐old rats, while the expression of Nestin decreased (Figure 3A–C). These findings suggest that the hippocampal homogenate supernatants of 20‐month‐old rats induce apoptosis and senescence in NPSCs. We used immunofluorescent staining to detect astrocytes, microglia, and neurons in the hippocampus of both 4‐week‐old and 20‐month‐old rats. The results showed that compared to 4‐week‐old rats, there were more astrocytes and microglia and fewer neurons in the hippocampus of 20‐month‐old rats (Figure S2A,B). To determine which cells regulate the senescence of NPSCs in the hippocampal microenvironment of 20‐month‐old rats, we co‐cultured primary NPSCs with H_2_O_2_‐treated microglia, astrocytes, and neurons (Figure 3D). Our results demonstrated that, compared with co‐cultured H_2_O_2_‐treated microglia and neurons, the proliferation ability of NPSCs co‐cultured with H_2_O_2_‐treated astrocytes was significantly decreased (Figure 3E,F), and senescence was significantly increased (Figure 3G,H). In addition, qRT‐PCR analysis showed that the expression of NPSC markers (Nestin and Sox2) was significantly decreased (Figure 3I,J). Overall, these findings demonstrate that H_2_O_2_‐treated astrocytes contribute to the senescence and apoptosis of NPSCs.

### 3.3 CSF‐1 Secreted by H_2_O_2_ ‐Treated Astrocytes Leads to the Blockade of NPSC Differentiation Into Neurons and Increased Cell Death

**FIGURE 4:**
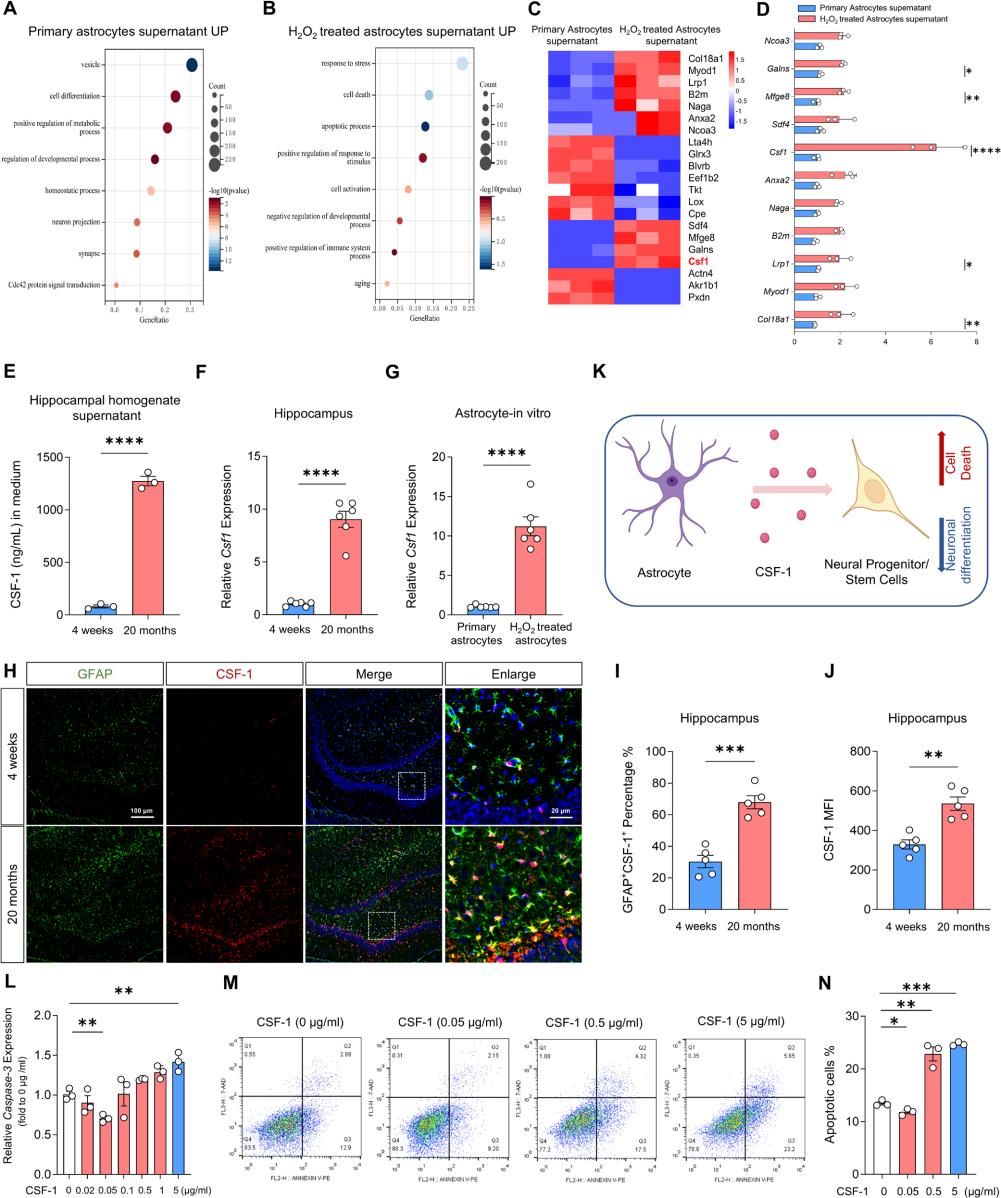
Selection and identification of CSF‐1. (A) GO analysis of related biological functions of up‐regulated genes in the supernatant of primary astrocytes. (B) GO analysis of related biological functions of up‐regulated genes in the supernatant of H_2_O_2_‐treated astrocytes. (C) Heat map of protein profiles of the supernatant of primary and H_2_O_2_‐treated astrocytes. (D) qRT‐PCR verified the relative expression of *Col18a1, Myod1, Lrp1, B2m, Naga, Anxa2, Csf1, Sdf4, Mfge8, Galns, Ncoa3* in the supernatants of primary and H_2_O_2_‐treated astrocytes. *p < 0.05, **p < 0.01, ****p < 0.0001, H_2_O_2_‐treated astrocytes versus primary astrocytes. Two‐way ANOVA followed by Bonferroni's tests (*n* = 3). (E) ELISA analysis of the expression levels of CSF‐1 in the hippocampal homogenate supernatant from 20‐month‐old and 4‐week‐old rats. ****p < 0.0001, 20 months versus 4 weeks. Student's *t*‐test (*n* = 3). (F) qRT‐PCR detection of the expression of *Csf‐1* in the hippocampus. ****p < 0.0001, 20 months versus 4 weeks. Student's *t*‐test (*n* = 6). (G) qRT‐PCR detection of the expression of *Csf‐1* in astrocytes. ****p < 0.0001, H_2_O_2_‐treated astrocytes versus Primary astrocytes. Student's *t*‐test (*n* = 6). (H) Immunofluorescence images of CSF‐1 expression in sections of hippocampus tissue of 20‐month‐old and 4‐week‐old rats. (I) Quantitative analysis of the percentage of GFAP^+^/CSF‐1^+^ double‐stained cells in whole GFAP^+^ cells. ***p < 0.001, 20‐month‐old and 4‐week‐old rats. Student's *t*‐test (*n* = 5). (J) Quantitative analysis of the mean fluorescence intensity of CSF‐1. **p < 0.01, 20‐month‐old and 4‐week‐old rats. Student's *t*‐test (*n* = 5). (K) The diagram showed that CSF‐1 secreted by H_2_O_2_‐treated astrocytes leads to the block of NPSCs differentiation into neurons and increased cell death. (L) qRT‐PCR detection of the expression of Caspase‐3. **p < 0.01, 0.05 μg/mL versus 0 μg/mL, 5 μg/mL versus 0 μg/mL. One‐way ANOVA followed by Bonferroni's tests (*n* = 3). (M, N) The apoptosis of NPSCs cultured with different concentrations of CSF‐1 recombinant protein was analyzed and quantified by flow cytometry. *p < 0.05, ***p < 0.001, 0.05 μg/mL versus 0 μg/mL, 5 μg/mL versus 0 μg/mL. One‐way ANOVA followed by Bonferroni's tests (*n* = 3).

To further explore how primary and H_2_O_2_‐treated astrocytes affect the proliferation, differentiation, and apoptosis of NPSCs, we investigated the differences in protein composition between the supernatant of primary astrocytes and that of H_2_O_2_‐treated astrocytes using proteomic analysis. The results of our gene ontology (GO) analysis suggested that upregulated genes in the supernatant of primary astrocytes are involved in biological processes such as cell differentiation, positive regulation of metabolic processes, and homeostasis (Figure 4A). In contrast, upregulated genes in the supernatant of H_2_O_2_‐treated astrocytes were associated with biological processes, such as cell death, apoptosis, and negative regulation of developmental processes (Figure 4B).

The heatmap of differentially expressed proteins between the supernatant of primary astrocytes and that of H_2_O_2_‐treated astrocytes is displayed in Figure 4C. We performed qRT‐PCR validation on the 11 proteins that showed high expression levels from the supernatant of H_2_O_2_‐treated astrocytes in the mass spectrometry analysis. The results showed that CSF‐1 was the most significantly differentially expressed protein in the supernatant of primary astrocytes and that of H_2_O_2_‐treated astrocytes (Figure 4D). Given that CSF‐1 is a secretory protein, we employed ELISA to analyze the expression levels of CSF‐1 in hippocampal homogenate supernatants from both 4‐ week‐old and 20‐month‐old rats. The results showed that CSF‐1 expression was significantly higher in the 20‐month group compared to the 4‐week group (Figure 4E). We used qRT‐PCR to analyze *Csf‐1* expression in the hippocampus of 4‐week‐old and 20‐month‐old rats. The results showed that CSF‐1 expression was significantly upregulated in the 20‐month group compared to the 4‐week group (Figure 4F). We further confirmed that CSF‐1 is primarily secreted by astrocytes in the senescent CNS through both in vitro and in vivo experiments. In vitro, we employed ELISA to analyze the expression levels of CSF‐1 in H_2_O_2_‐treated astrocytes, microglia, and neurons. The results showed that CSF‐1 expression was significantly higher in H_2_O_2_‐treated astrocytes compared to both H_2_O_2_‐treated microglia and neurons (Figure S4A). We also employed qRT‐PCR to analyze the expression levels of *Csf‐1* in primary and H_2_O_2_‐treated astrocytes. The results showed that CSF‐1 expression was higher in H_2_O_2_‐treated astrocytes compared to primary astrocytes (Figure 4G). In vivo, we performed immunofluorescence staining to detect the expression of CSF‐1 in astrocytes, microglia, and neurons in the hippocampus of 20‐month‐old rats. The results indicated that the number of GFAP^+^/CSF‐1^+^ double‐stained cells was significantly higher than that of IBA1^+^/CSF‐1^+^ double‐stained cells and NeuN^+^/CSF‐1^+^ double‐stained cells (Figure S4B). We also used immunofluorescence staining to detect the expression of CSF‐1 in astrocytes in the hippocampus of 4‐week‐old and 20‐month‐old rats. The results indicated that the percentage of GFAP^+^/CSF‐1^+^ double‐stained cells was significantly higher in the 20‐month group than in the 4‐week group (Figure 4H–J). Taken together, our in vivo and in vitro results demonstrated that astrocytes are the major supplier of CSF‐1 in the senescent CNS. Overall, we speculated that H_2_O_2_‐treated astrocytes regulate CSF‐1 to block the differentiation of NPSCs into neuronal cells and promote cell death (Figure 4K). To test this hypothesis, we isolated and cultured NPSCs in vitro by adding different concentrations of CSF‐1 recombinant protein to determine its effects on NPSCs. The qRT‐PCR and immunofluorescence staining results showed that compared to the control group (0 μg/mL CSF‐1), the proliferation level of NPSCs decreased in the 5 μg/mL CSF‐1 group, as indicated by Ki67 expression. However, as the CSF‐1 concentration increased, the proliferation level of NPSCs decreased. The proliferation level of NPSCs was lowest in the 5 μg/mL CSF‐1 group (Figure S5A–C). Furthermore, the flow cytometry results showed that the proliferation rate of NPSCs was highest in the 0.05 μg/mL CSF‐1 group and lowest in the 5 μg/mL CSF‐1 group (Figure S5D,E). Additionally, as shown in Figure S6A,B, immunofluorescence staining results showed that compared with the 0 μg/mL CSF‐1 group, the Tuj1^+^ cells of NPSCs increased in the 0.05 μg/mL CSF‐1 group. However, as the CSF‐1 concentration increased, the differentiation of NPSCs into neurons decreased. Notably, the Tuj1^+^ cell count was lowest in the 5 μg/mL CSF‐1 group. In conclusion, an appropriate low concentration (0.05 μg/mL) of CSF‐1 promoted the differentiation of NPSCs into neuronal cells, whereas a pathologically high concentration (5 μg/mL) of CSF‐1 inhibited this process. Moreover, qRT‐PCR and flow cytometry results showed that compared with the 0 μg/mL CSF‐1 group, the apoptosis rate of NPSCs was lowest in the 0.05 μg/mL CSF‐1 group and highest in the 5 μg/mL CSF‐1 group (Figure 4L–N). In summary, these results suggest that an appropriate low concentration (0.05 μg/mL) of CSF‐1 can enhance the proliferation and differentiation of NPSCs and inhibit apoptosis, whereas a pathologically high concentration (5 μg/mL) of CSF‐1 can inhibit the proliferation and differentiation of NPSCs and induce apoptosis.

### 3.4 Selection of miRNAs Upstream of CSF‐1

**FIGURE 5:**
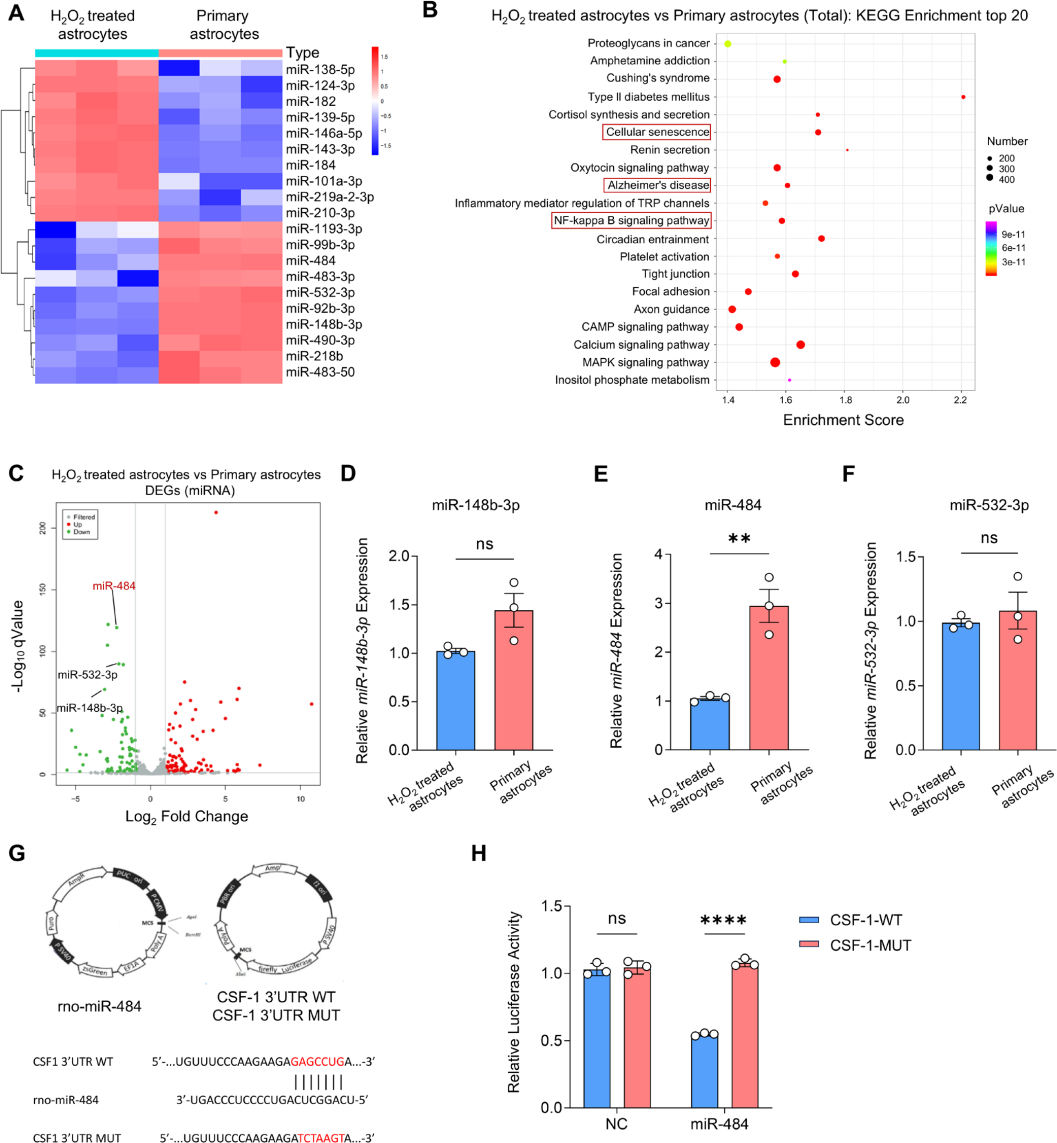
Selection and identification of miRNAs upstream of CSF‐1. (A) Heat map of miRNA sequencing in primary and H_2_O_2_‐treated astrocytes. (B) KEGG analysis of relevant pathways. (C) Volcano plot of differentially expressed miRNAs. (D–F) qRT‐PCR detection of the expression of miR‐148b‐3p, miR‐484, and miR‐532‐3p. **p < 0.01, H_2_O_2_‐treated astrocytes versus Primary astrocytes. Student's *t*‐test (*n* = 3). (G) Diagram of the structure and core fragment of the luciferase reporter plasmid. (H) Quantitative analysis of luciferase reporter. ****p < 0.0001, CSF‐1‐WT versus CSF‐1‐MUT. Two‐way ANOVA followed by Bonferroni's tests (*n* = 3).

miRNA activity can modulate NPSC proliferation and neuronal differentiation in both rats and mice [23, 32, 33]. To identify the master regulators of CSF‐1‐directed proliferation and differentiation of NPSCs, we prepared primary astrocytes and H_2_O_2_‐treated astrocytes for miRNA sequencing. A total of 503 miRNAs were identified, and 124 miRNAs with a fold change > 1.5 and *p*‐values (Student's *t*‐test) < 0.05 were considered to be differentially expressed miRNAs. The cluster heat map and volcano plots (Figure 5A,C) revealed a fold change greater than 1.5 in the differentially expressed miRNAs between the primary and H_2_O_2_‐treated astrocytes. Following this, we performed KEGG pathway analysis based on RNA‐seq profiles and found that the predicted target genes of differentially expressed miRNAs were correlated with the cellular senescence and neurodegenerative diseases pathways (Figure 5B). Next, MirDateBase and TargetScan were used to predict possible upstream miRNAs of CSF‐1. The MirDateBase predicted 26 miRNAs, while TargetScan predicted 259 miRNAs.

Differentially expressed miRNAs in primary and H_2_O_2_‐treated astrocytes were cross‐referenced with those predicted to target CSF‐1 by these online databases (Figure S7A). The data showed that the three miRNAs containing conserved target sites for CSF‐1 could serve as upstream targets of CSF‐1 (Figure S7B). We then used qRT‐PCR to verify these three candidate molecules. The qRT‐PCR results demonstrated that the difference in miR‐484 expression was most significant between the primary and H_2_O_2_‐treated astrocytes (Figure 5D–F). Additionally, we constructed dual‐luciferase reporter plasmids for both wild‐type and mutant forms of CSF‐1 (Figure 5G). Through a dual‐luciferase reporter assay, we observed that transfection with miR‐484 mimics led to a significant decrease in the luciferase activity of the wild‐type CSF‐1 reporter gene. In contrast, the luciferase activity of the mutant CSF‐1 reporter gene remained unaffected (Figure 5H). These findings indicate that miR‐484 directly targets CSF‐1.

### 3.5 miR‐484 Regulates NPSC Proliferation and Differentiation Into Neurons by Targeting CSF‐1

**FIGURE 6:**
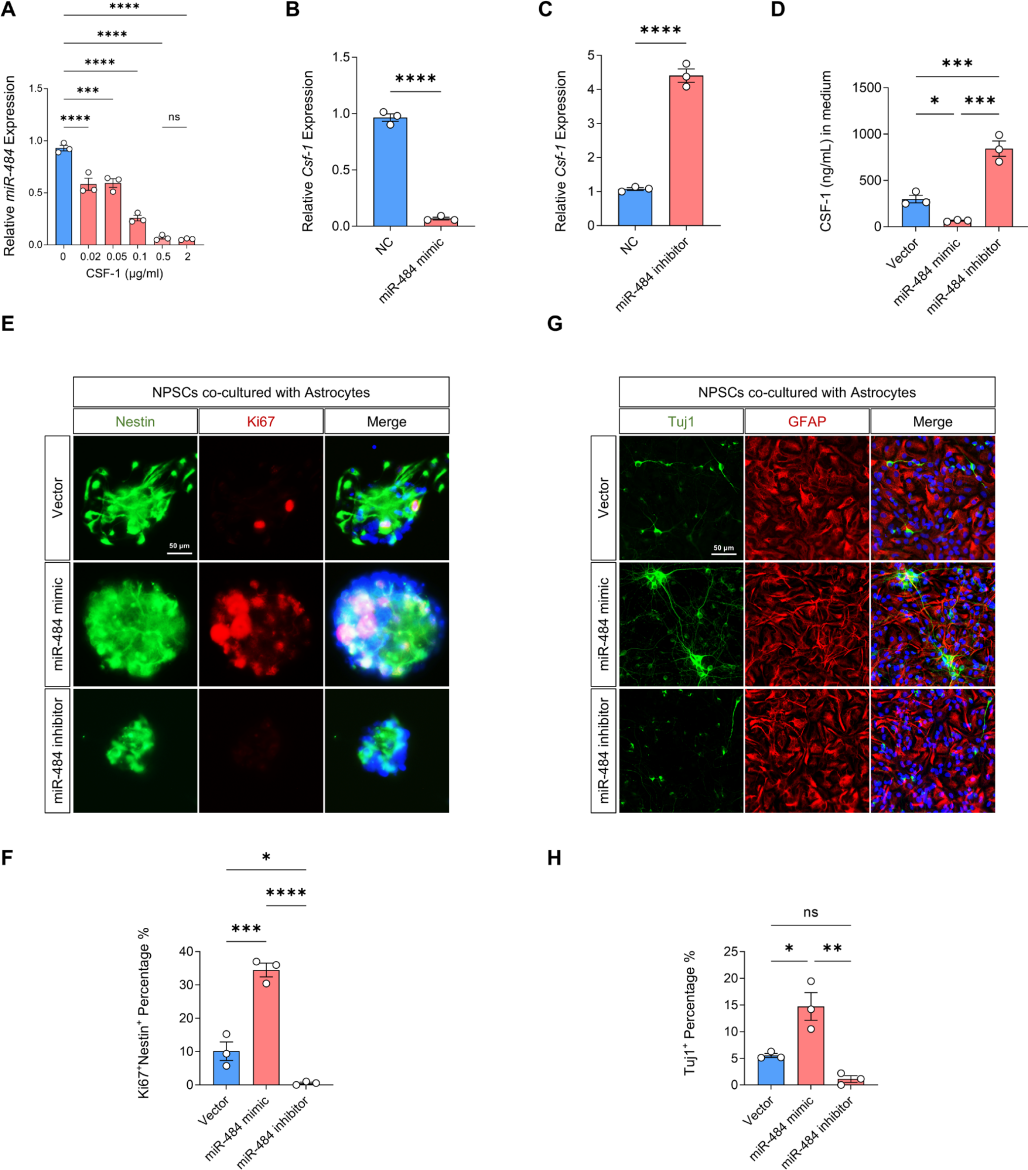
Effect of miR‐484 on proliferation and differentiation of NPSCs. (A) qRT‐PCR detection of the expression of miR‐484. ***p < 0.001, ****p < 0.0001, 0.02 μg/mL versus 0 μg/mL, 0.05 μg/mL versus 0 μg/mL, 0.1 μg/mL versus 0 μg/mL, 0.5 μg/mL versus 0 μg/mL, 2 μg/mL versus 0 μg/mL. One‐way ANOVA followed by Bonferroni's tests (*n* = 3). (B, C) qRT‐PCR detection of the expression of *Csf‐1*. ****p < 0.0001, miR‐484 mimic versus NC, miR‐484 inhibitor versus NC. Student's *t*‐test (*n* = 3). (D) ELISA analysis of the expression levels of CSF‐1. *p < 0.05, ***p < 0.001, miR‐484 mimic versus Vector, miR‐484 inhibitor versus Vector, miR‐484 inhibitor versus miR‐484 mimic. One‐way ANOVA followed by Bonferroni's tests (*n* = 3). (E) Immunofluorescence images of Ki67 expression in NPSCs after co‐culture with miR‐484 treated astrocytes. (F) Quantitative analysis of the percentage of Nestin^+^/Ki67^+^ double‐stained cells in whole Nestin^+^ cells. *p < 0.05, ***p < 0.001, ****p < 0.0001, miR‐484 mimic versus Vector, miR‐484 inhibitor versus Vector, miR‐484 inhibitor versus miR‐484 mimic. One‐way ANOVA followed by Bonferroni‐s tests (*n* = 3). (G) Immunofluorescence images of Tuj1 expression in NPSCs after co‐culture with miR‐484 treated astrocytes. (H) Quantitative analysis of the percentage of Tuj1^+^ cells in whole cells. *p < 0.05, **p < 0.01, miR‐484 mimic versus Vector, miR‐484 inhibitor versus Vector, miR‐484 inhibitor versus miR‐484 mimic. One‐way ANOVA followed by Bonferroni's tests (*n* = 3).

miR‐484, which is abundant in the CNS, is an important regulator of gene expression, playing a significant role in various cellular processes at the transcriptional level. Therefore, we hypothesized that miR‐484 could regulate the function of NPSCs. To explore the effect of miR‐484 on the proliferation and differentiation of NPSCs, we transfected an miR‐484 mimic and miR‐484 inhibitor into H_2_O_2_‐treated astrocytes and then co‐cultured with NPSCs. The proliferation level of NPSCs increased after co‐culture with H_2_O_2_‐treated astrocytes transfected with miR‐484 mimic and decreased after co‐culture with H_2_O_2_‐treated astrocytes transfected with miR‐484 inhibitor, as assessed by Ki67 expression (Figure 6E,F). The differentiation level of NPSCs also increased after co‐culture with astrocytes transfected with miR‐484 mimic and decreased after co‐culture with astrocytes transfected with an miR‐484 inhibitor, as assessed by Tuj1 expression (Figure 6G,H). qRT‐PCR results showed that compared with the 0 μg/mL CSF‐1 group, as the CSF‐1 concentration increased, the expression of miR‐484 decreased. Additionally, when compared to the NC group, the expression of CSF‐1 was reduced in the miR‐484 mimic group, whereas it was elevated in the miR‐484 inhibitor group (Figure 6A–D). Overall, these results indicate that miR‐484 promotes NPSC proliferation and differentiation into neurons by targeting CSF‐1.

### 3.6 miR‐484 Overexpression Improved Cognitive Function in 20‐Month‐Old Rats, Promoted NPSC Proliferation, and Reduced Apoptosis

**FIGURE 7:**
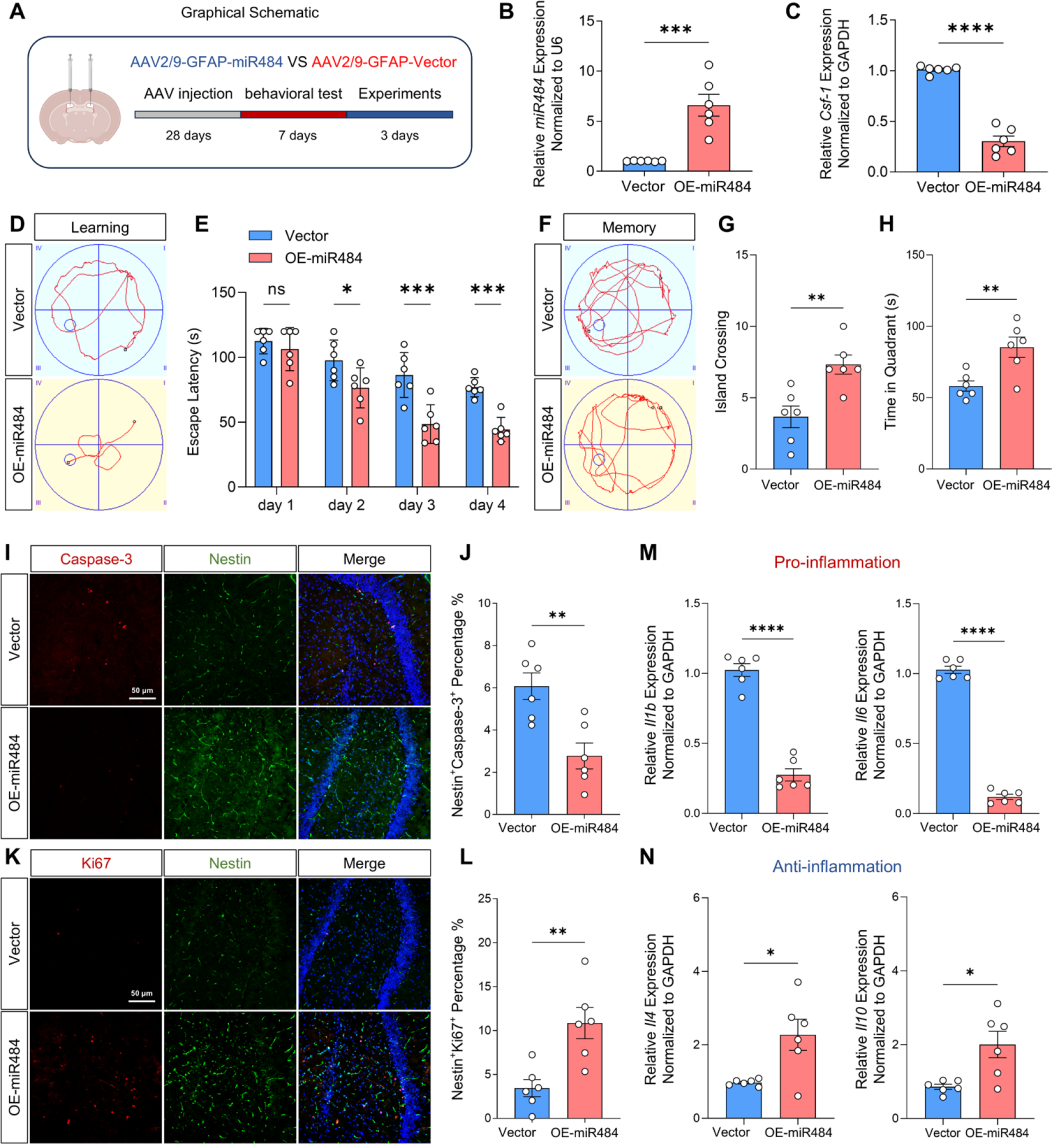
miR‐484 overexpression promoted the recovery of NPSCs in vivo. (A) The graphical schematic of the in vivo experiments. (B, C) qRT‐PCR detection of the expression of miR‐484 and *Csf‐1*. ***p < 0.001, ****p < 0.0001, OE‐miR484 versus Vector. Student's *t*‐test (*n* = 6). (D) Representative results of the Morris water maze test during the learning period for 20‐month‐old rats treated with OE‐miR484. (E) Quantitative statistics of escape latency in rats. *p < 0.05, ***p < 0.001, OE‐miR484 versus Vector. Two‐way ANOVA followed by Bonferroni's tests (*n* = 6). (F) Representative results of the Morris water maze test during the memory period for 20‐month‐old rats treated with OE‐miR484. (G, H) Quantitative statistics of number of crossing island and dwell time in the target quadrant in rats. **p < 0.01, OE‐miR484 versus Vector. Student's *t*‐test (*n* = 6). (I) Immunofluorescence images of Nestin^+^/Caspase‐3^+^ double‐stained cells in the hippocampus. (J) Quantitative analysis of the percentage of Nestin^+^/Caspase‐3^+^ double‐stained cells in whole cells in the hippocampus. **p < 0.01, OE‐miR484 versus Vector. Student's *t*‐test (*n* = 6). (K) Immunofluorescence images of Nestin^+^/Ki67^+^ double‐stained cells in the hippocamus. (L) Quantitative analysis of the percentage of Nestin^+^/Ki67^+^ double‐stained cells in whole cells in the hippocampus. **p < 0.01, OE‐miR484 versus Vector. Students's *t*‐test (*n* = 6). (M, N) qRT‐pCR detection of the expression of *Il‐1β*, *Il‐6*, *Il‐4* and *Il‐10*. *p < 0.05, ****p < 0.0001, OE‐miR484 versus Vector. Student's *t*‐test (*n* = 6).

We further explored the effect of miR‐484 on NPSC function in vivo by injecting AAV2/9‐GFAP‐miR‐484 into the hippocampi of 20‐month‐old rats (Figure 7A). Changes in cognitive function were assessed through behavioral experiments conducted 28 days post‐injection. Subsequently, miR‐484 expression in the hippocampus was measured using qRT‐PCR. The findings indicated that, compared to the vector group, miR‐484 expression was elevated, while CSF‐1 expression was reduced in the hippocampus following the injection of AAV2/9‐GFAP‐miR484 (Figure 7B,C). We detected the CSF‐1 expression performed from AAV2/9‐GFAP‐miR‐484 transfected animal brains by immunofluorescent staining. The results showed that CSF‐1 expression was significantly downregulated in the AAV2/9‐GFAP‐miR‐484 transfected brains (Figure S8). The MWM was utilized to evaluate spatial learning (Figure 7D) and memory (Figure 7F) in 20‐month‐old rats. The results showed that during the learning phase, escape latency decreased in the miR‐484 overexpression group compared to that in the vector group (Figure 7E). Furthermore, in the memory phase, the miR‐484 overexpression group exhibited an increased number of island crossings (Figure 7G) and longer dwell time in the target quadrant (Figure 7H) compared to the vector group.

Immunofluorescence analysis revealed that miR‐484 overexpression reduced apoptosis and enhanced the proliferation of NPSCs, as evidenced by Caspase‐3 and Ki67 expression levels (Figure 7I–L). Moreover, miR‐484 overexpression in astrocytes resulted in diminished proinflammatory effects and increased anti‐inflammatory effects (Figure 7M,N), leading to decreased inflammation in the nervous system and aiding in cognitive function recovery. In summary, these results demonstrate that the overexpression of miR‐484 improves learning and memory function in 20‐month‐old rats and promotes the proliferation of NPSCs, reducing apoptosis.

## 4 Discussion

With aging, impairment of hippocampal neurogenesis frequently leads to age‐related neurodegenerative diseases and causes cognitive dysfunctions [34]. During the process of aging, the decline in adult neurogenesis is primarily attributed to the diminished proliferative and differentiative capacity of NSCs [5], which are significantly influenced by the neural microenvironment [7]. However, the specific mechanisms by which the neural microenvironment regulates NSC differentiation into neurons remain unclear. In this study, miR‐484 was found to regulate the secretion of CSF‐1 in H_2_O_2_‐treated astrocytes, promoting the proliferation of NPSCs and their differentiation into neurons, ultimately improving cognitive function in 20‐month‐old rats. Our results demonstrated that the decline in learning and memory abilities in 20‐month‐old rats was associated with the depletion of hippocampal NPSCs, which was initially consistent with previous studies [23, 27]. To elucidate the specific mechanisms by which the hippocampal microenvironment regulates NPSC function, we performed co‐cultures involving H_2_O_2_‐treated microglia, astrocytes, and neurons with NPSCs. The results indicated a notable decrease in the proliferation capacity of NPSCs co‐cultured with H_2_O_2_‐treated astrocytes, along with a pronounced increase in senescence. Similarly, primary astrocytes regulate the proliferation and differentiation of adult hippocampal NSCs, as well as the maturation of newly formed neurons [35]. Importantly, in the CNS, different types of proteins are produced and secreted by astrocytes to remodel peripheral and synaptic extracellular matrices [36]. Building on this, we performed mass spectrometry analysis and confirmed that multiple proteins were differentially expressed between the supernatants of primary and H_2_O_2_‐treated astrocytes. Of particular interest, the expression of CSF‐1 was significantly higher in H_2_O_2_‐treated astrocytes.

Previous studies have indicated that CSF‐1 can control the proliferation and differentiation of mononuclear phagocyte cells, such as microglia, through self‐regulation [37, 38]. However, the specific role of CSF‐1 in NPSC senescence remains unclear. In this study, we cultured NPSCs in vitro with different concentrations of CSF‐1 recombinant protein. We found that high concentrations of CSF‐1 inhibited the proliferation and differentiation of NPSCs and promoted their apoptosis. In contrast, low concentrations of CSF‐1 enhanced the proliferation and differentiation of NPSCs and inhibited their apoptosis. Our research findings are consistent with previous studies [39, 40, 41]. Thus, the maintenance of the Ki67^+^ ratio in NPSCs co‐cultured with H_2_O_2_‐treated microglia or neurons may be attributed to the low expression of CSF‐1 in both H_2_O_2_‐treated microglia and neurons, as indicated by ELISA (Figure S4A). Indeed, CSF‐1R inhibitors have been shown to mitigate neuroinflammation, enhance the survival and proliferative capabilities of microglia, and impede the advancement of Alzheimer's disease in mouse models [42]. Furthermore, CSF‐1R inhibitors have shown potential in reducing neuroinflammation and improving cognitive function in models of Alzheimer's disease [43, 44] and Parkinson's disease [45]. In summary, H_2_O_2_‐treated astrocytes secrete high concentrations of CSF‐1, leading to increased apoptosis and decreased hippocampal neurogenesis, ultimately resulting in cognitive impairment in 20‐month‐old rats.

NPSCs express a distinct set of miRNAs that are crucial for preserving both the self‐renewal capacity and differentiation processes [46]. For instance, miR‐132 has been demonstrated to inhibit the differentiation and proliferation of NSCs by releasing proinflammatory cytokines [47], whereas suppressing miR‐219 has been shown to enhance NSC proliferation [48]. In another study, miR‐181a‐5p was found to facilitate NSC proliferation and improve the learning and memory functions of aged mice [32]. Previous studies have reported that the target genes of miR‐484 are primarily associated with genes related to cognitive function. The upregulation of miR‐484 in cortical and cerebellar regions may be linked to changes in cognitive, motor, and emotional behaviors in mice [49, 50, 51]. However, the specific regulatory mechanism of miR‐484 in the nervous system has not been thoroughly investigated. In the present study, overexpression of miR‐484 led to an increase in the proliferation and neuronal differentiation of NPSCs, resulting in improved cognitive function in 20‐month‐old rats and a significant downregulation of CSF‐1 expression. In addition, mRNA expression profiling, bioinformatics analysis, and dual‐luciferase reporter gene assays revealed the potential of CSF‐1 to serve as a direct target of miR‐484. This indicates that miR‐484 can promote NPSC neurogenesis by regulating CSF‐1.

While our study provides valuable insights, its limitations should be noted. First, the regulatory mechanism of downstream signaling pathways mediated by CSF‐1 requires further exploration. Second, it remains to be determined whether miR‐484 exerts its effects through the modulation of other target genes.

## 5 Conclusion

In this study, we found that high concentrations of CSF‐1 inhibited the proliferation and differentiation of NPSCs while promoting their apoptosis. Furthermore, we identified an interaction between miR‐484 and the 3'‐UTR of CSF‐1. Notably, our findings demonstrated that miR‐484 can significantly regulate the proliferation, differentiation, and apoptosis of NPSCs by targeting CSF‐1, which provides a novel therapeutic approach for improving age‐related nervous system dysfunction in clinical practice.

## Author Contributions

Yuejuan Ling, Qianqian Liu, and Wei Shi: conceptualization. Jiahua Qu and Zhichao Lu: methodology. Jiahua Qu, Zhichao Lu, Yongbo Cheng, and Song Deng: validation. Jiahua Qu, Zhichao Lu, and Qianqian Liu: formal analysis. Jiahua Qu and Yuejuan Ling: writing – original draft preparation. Yuejuan Ling and Wei Shi: writing – review and editing. Jiahua Qu, Zhichao Lu, Yongbo Cheng, and Qianqian Liu: visualization. Yuejuan Ling and Wei Shi: supervision. All authors have read and agreed to the published version of the manuscript.

## Ethics Statement

All the experimental procedures were performed with the approval of the Experimental Animal Welfare Ethics Committee of Nantong University (IACUC) (No. S20240116‐012) and followed the Institutional Animal Care and Use Committee guidelines.

## Conflicts of Interest

The authors declare no conflicts of interest.

## Supporting information


Appendix S1.

## Data Availability

The data supporting this study's findings are available from the corresponding author upon reasonable request.
