# Supplementary material for: miR‐484 in Hippocampal Astrocytes of Aged and Young Rats Targets CSF‐1 to Regulate Neural Progenitor/Stem Cell Proliferation and Differentiation Into Neurons
Source: CNS Neurosci Ther. 2025 Apr 30;31(5):e70415. doi: 10.1111/cns.70415 (PMC12042212; doi:10.1111/cns.70415)
Supplement: Supplementary file 1 — Appendix S1. [file CNS-31-e70415-s001.docx]

**Supplementary materials for**

**miR-484 in Hippocampal Astrocytes of Aged and Young Rats Targets CSF-1 to Regulate Neural Progenitor/Stem Cell Proliferation and Differentiation into Neurons**

Jiahua Qu^1#^, Zhichao Lu^1#^, Yongbo Cheng^1^, Song Deng^1^, Wei Shi^1*^, Qianqian Liu^1*^, Yuejuan Ling^1,2*^

^1^Research Center of Clinical Medicine, Co-innovation Department of Neurosurgery, Affiliated Hospital of Nantong University, Medical School of Nantong University, Nantong, Jiangsu 226001, China

^2^Institute of Pain Medicine and Special Environmental Medicine, Nantong University, Nantong, Jiangsu 226001, China

^#^Jiahua Qu and Zhichao Lu contributed equally to this work.

***Correspondence**

Yuejuan Ling: lingyuejuan@163.com

Qianqian Liu: ntfyliuqianqian@163.com

Wei Shi: [fysw@ntu.edu.cn](mailto:fysw@ntu.edu.cn)

**Supplementary Figures**


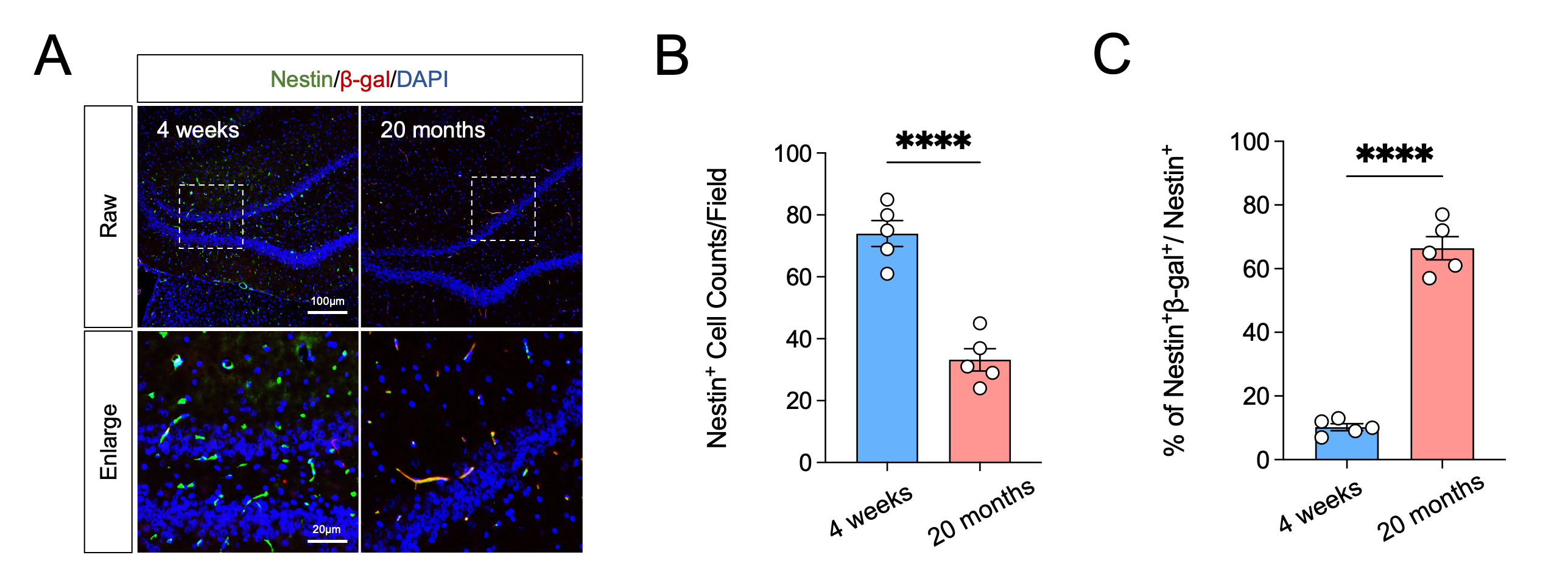


**Supplementary Figure 1. Identification of senescent NPSCs in the hippocampus.** (A) Immunofluorescence images of Nestin^+^/β-gal^+^ double-stained cells in the hippocampus. (B-C) Quantitative analysis of the number of Nestin^+^ cells and the percentage of Nestin^+^/β-gal^+^ double-stained cells in Nestin^+^ cells. *****p* < 0.0001, 20 months versus 4 weeks. Student's *t*‐test (*n* = 5).


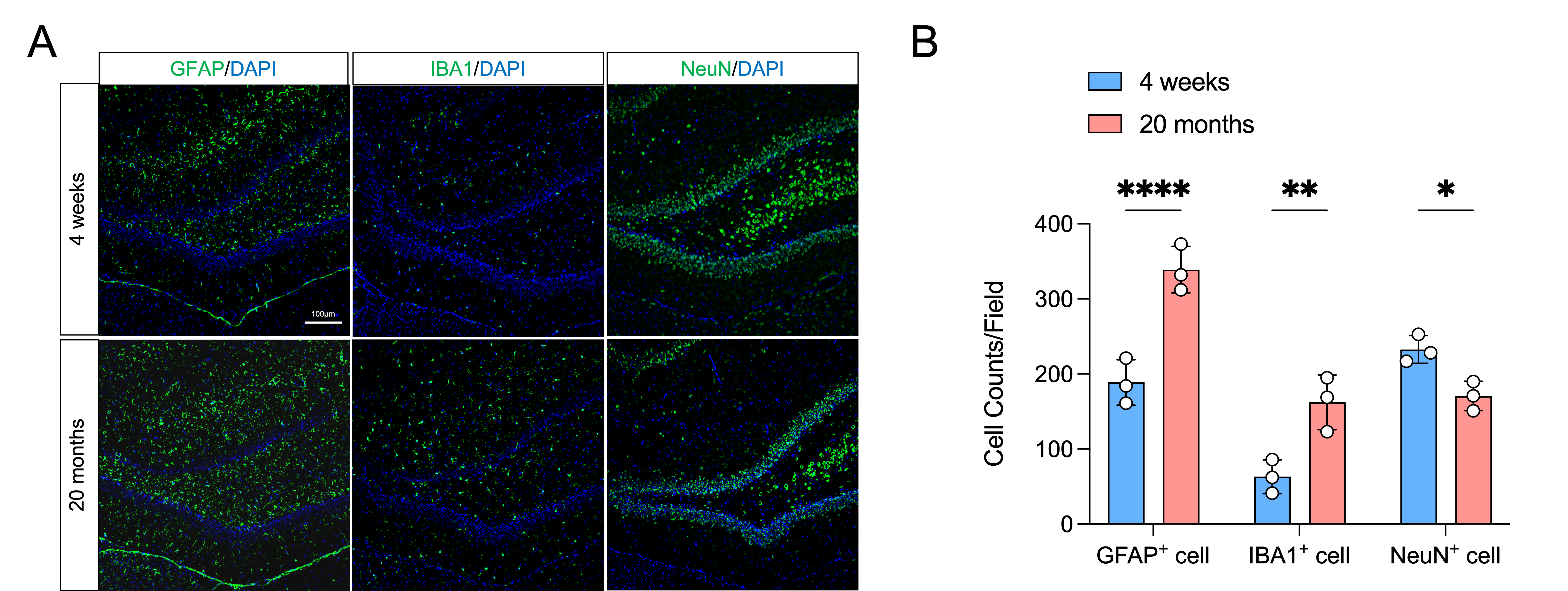


**Supplementary Figure 2. Astrocytes, microglia, and neurons in the hippocampus of both 4 weeks old and 20 months old rats.** (A) Immunofluorescence images of GFAP^+^, IBA1^+^ and NeuN^+^ cells in the hippocampus. (B) Quantitative analysis of the number of GFAP^+^, IBA1^+^ and NeuN^+^ cells. **p* < 0.05, ***p* < 0.01, *****p* < 0.0001, 20 months versus 4 weeks. Two‐way ANOVA followed by Bonferroni's tests (*n* = 3).


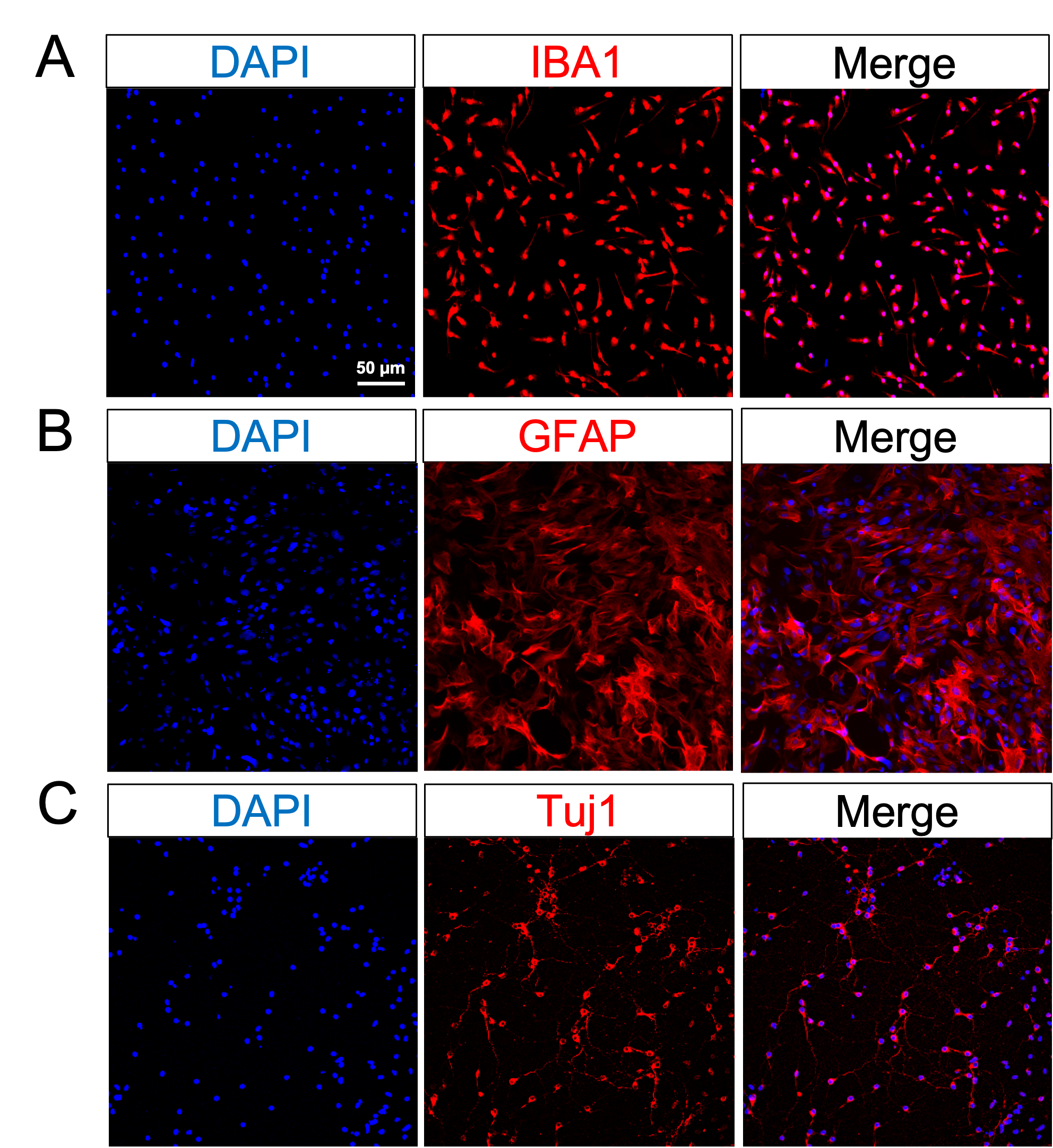


**Supplementary Figure 3. Identification of primary astrocytes, primary microglia, and primary neurons.** (A) Immunofluorescence identification images of primary microglia. (B) Immunofluorescence identification images of primary astrocytes. (C) Immunofluorescence identification images of primary neurons.


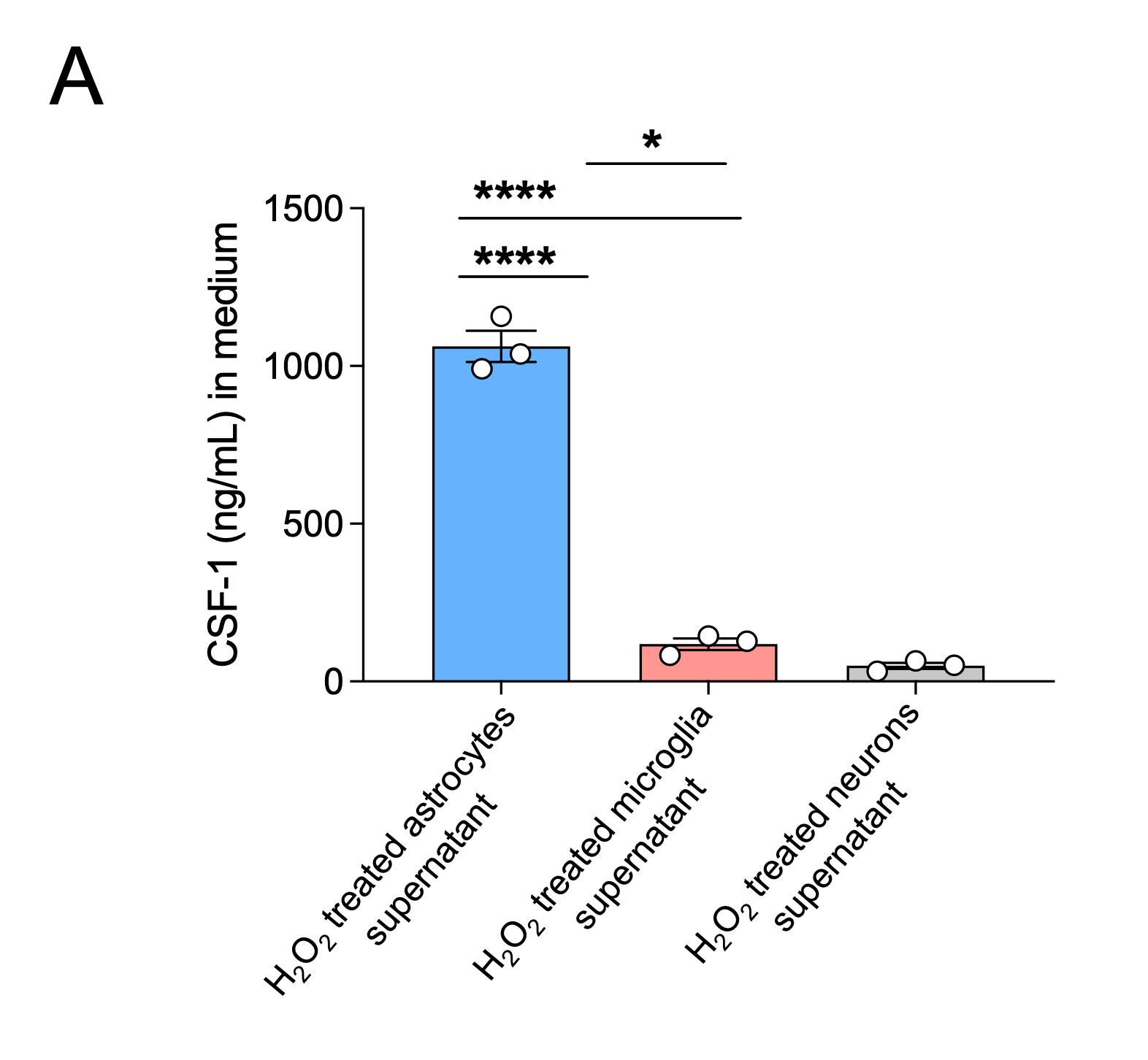


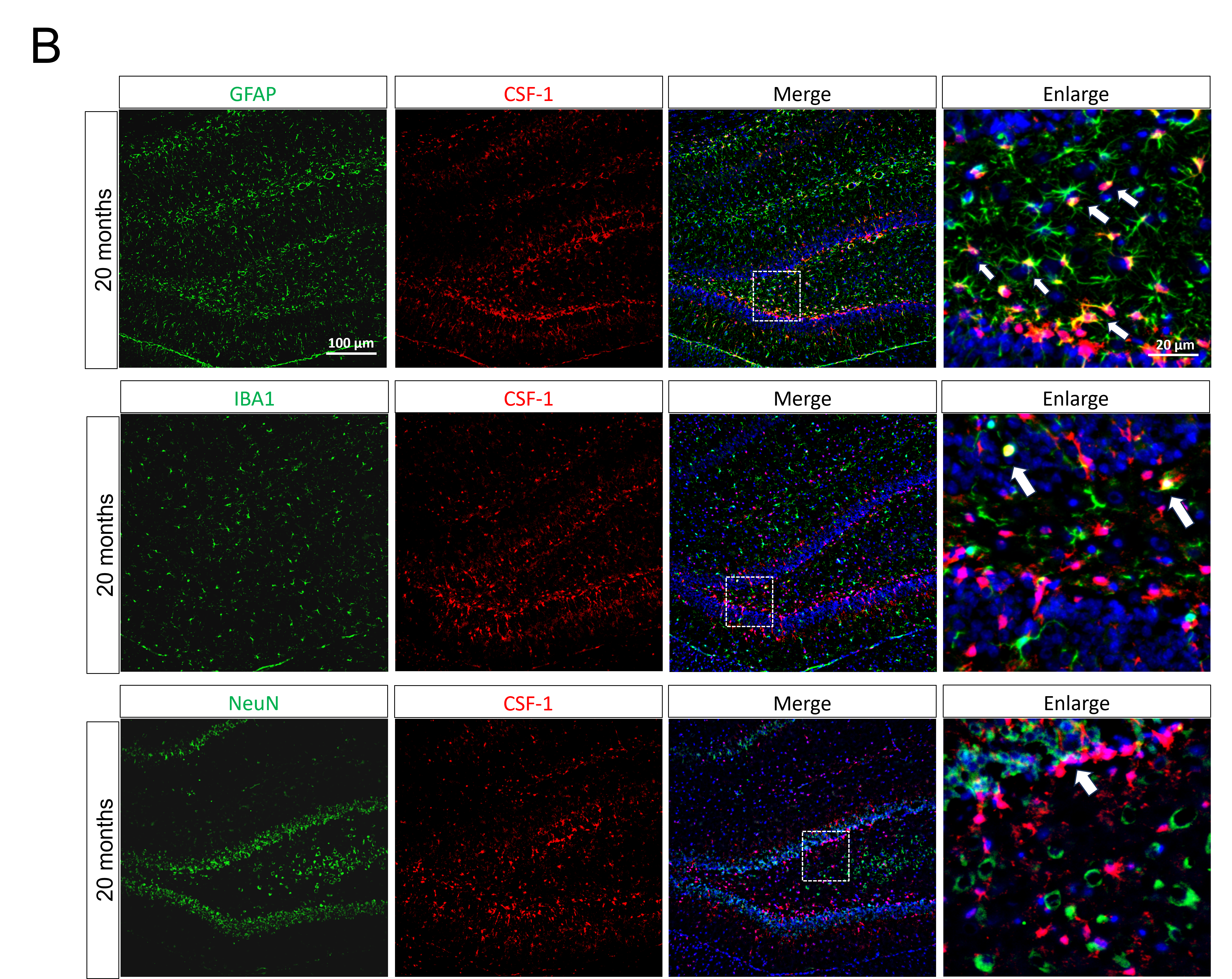


**Supplementary Figure 4. The expression level of CSF-1 in astrocytes, microglia and neurons.** (A) ELISA analysis of the expression level of CSF-1 in the supernatant of H_2_O_2_ treated astrocytes, H_2_O_2_ treated microglia, H_2_O_2_ treated neurons. **p* < 0.05, *****p* < 0.0001, H_2_O_2_ treated astrocytes supernatant versus H_2_O_2_ treated microglia supernatant, H_2_O_2_ treated astrocytes supernatant versus H_2_O_2_ treated neurons supernatant, H_2_O_2_ treated microglia supernatant versus H_2_O_2_ treated neurons supernatant. One‐way ANOVA followed by Bonferroni's tests (*n* = 3). (B) Immunofluorescence images of GFAP^+^/CSF-1^+^ double-stained cells, IBA1^+^/CSF-1^+^ double-stained cells and NeuN^+^/CSF-1^+^ double-stained cells in the hippocampus.


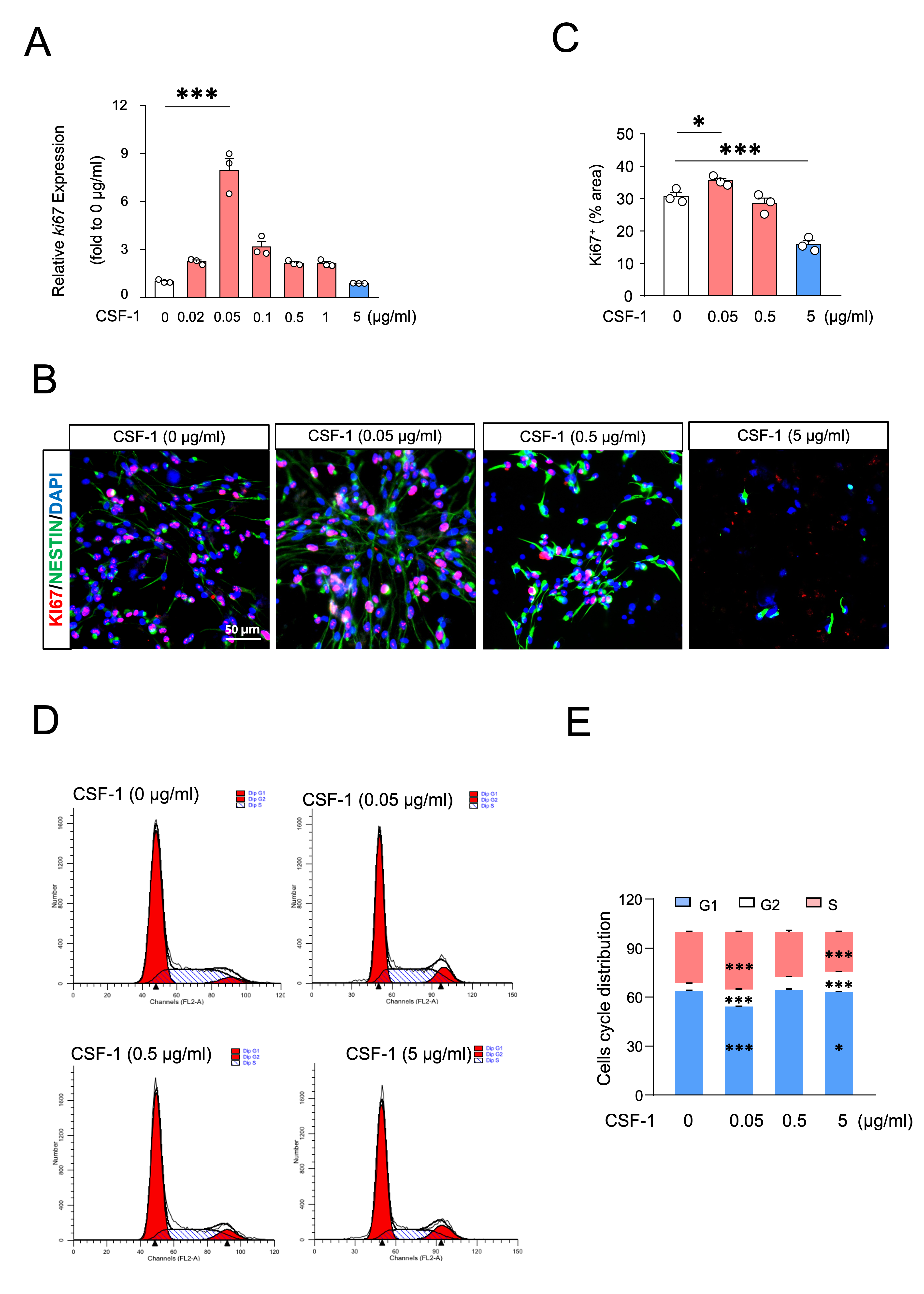


**Supplementary Figure 5. High concentration of CSF-1 inhibited the proliferation of NPSCs, while low concentrations of CSF-1 promote their proliferation.** (A) qRT-PCR detection of the expression of Ki67. ****p* < 0.001, 0.05 μg/ml versus 0 μg/ml. One‐way ANOVA followed by Bonferroni's tests (*n* = 3). (B) Immunofluorescence images of Ki67 expression in NPSCs after treatment with different concentrations of CSF-1 recombinant protein for 24 h. (C) Quantitative analysis of the percentage of Nestin^+^/Ki67^+^ double-stained cells in whole cells. **p* < 0.05, ****p* < 0.001, 0.05 μg/ml versus 0 μg/ml, 5 μg/ml versus 0 μg/ml. One‐way ANOVA followed by Bonferroni's tests (*n* = 3). (D-E) The proportion of cells in G1 phase, G2 phase and S phase after treatment with different concentrations of CSF-1 recombinant protein was analyzed and quantified by flow cytometry. G1 phase, **p* < 0.05, ****p* < 0.001, 0.05 μg/ml versus 0 μg/ml, 5 μg/ml versus 0 μg/ml. G2 phase, ****p* < 0.001, 0.05 μg/ml versus 0 μg/ml, 5 μg/ml versus 0 μg/ml. S phase, ****p* < 0.001, 0.05 μg/ml versus 0 μg/ml, 5 μg/ml versus 0 μg/ml. Two‐way ANOVA followed by Bonferroni's tests (*n* = 3).


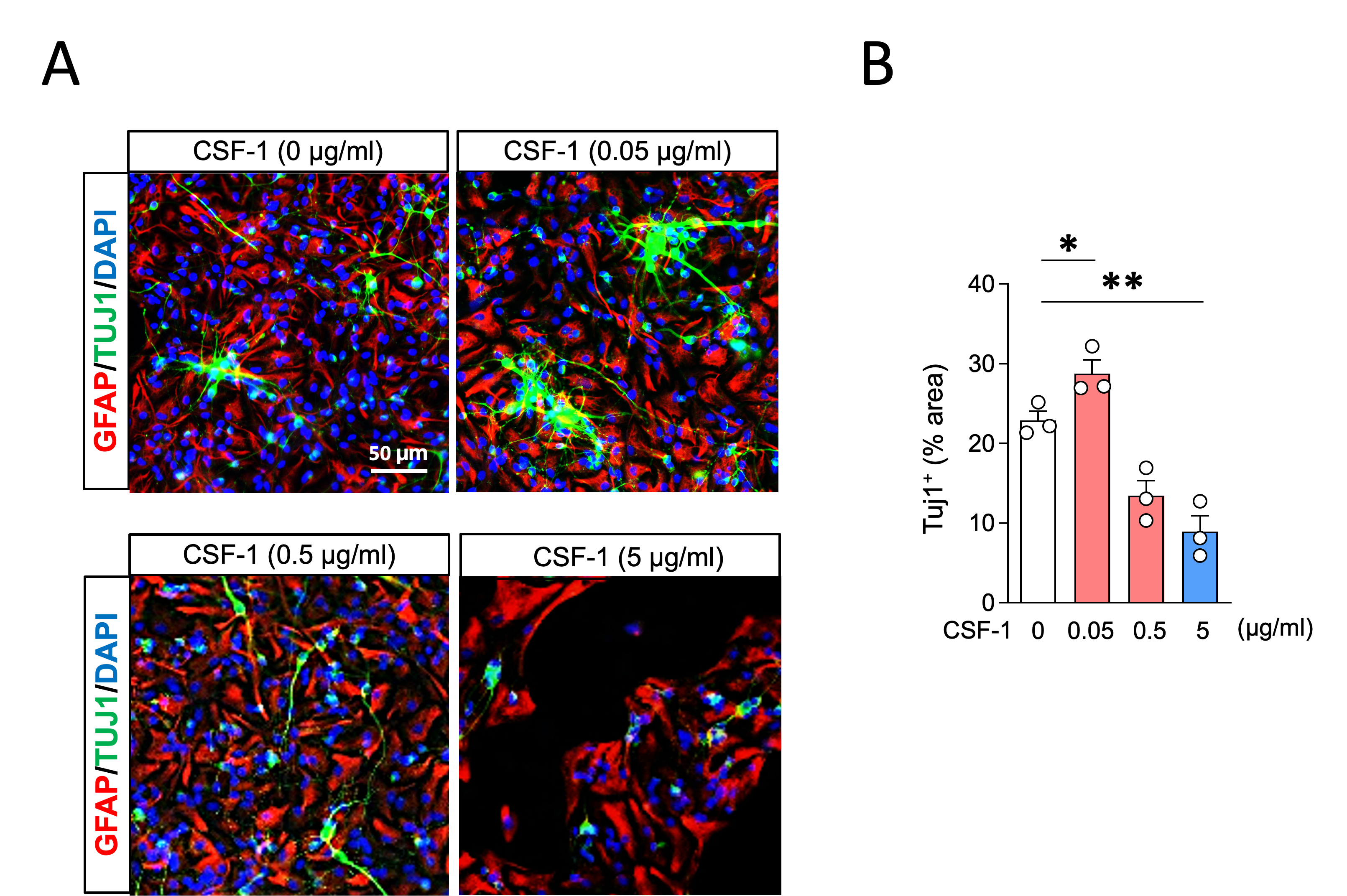


**Supplementary Figure 6. High concentration of CSF-1 inhibited the differentiation of NPSCs into neurons, while low concentrations of CSF-1 promote their differentiation.** (A) Immunofluorescence images of Tuj1 expression of NPSCs after treatment with different concentrations of CSF-1 recombinant protein. (B) Quantitative analysis of the percentage of Tuj1^+^ cells in whole cells. **p* < 0.05, ***p* < 0.01, 0.05 μg/ml versus 0 μg/ml, 5 μg/ml versus 0 μg/ml. One‐way ANOVA followed by Bonferroni's tests (*n* = 3).


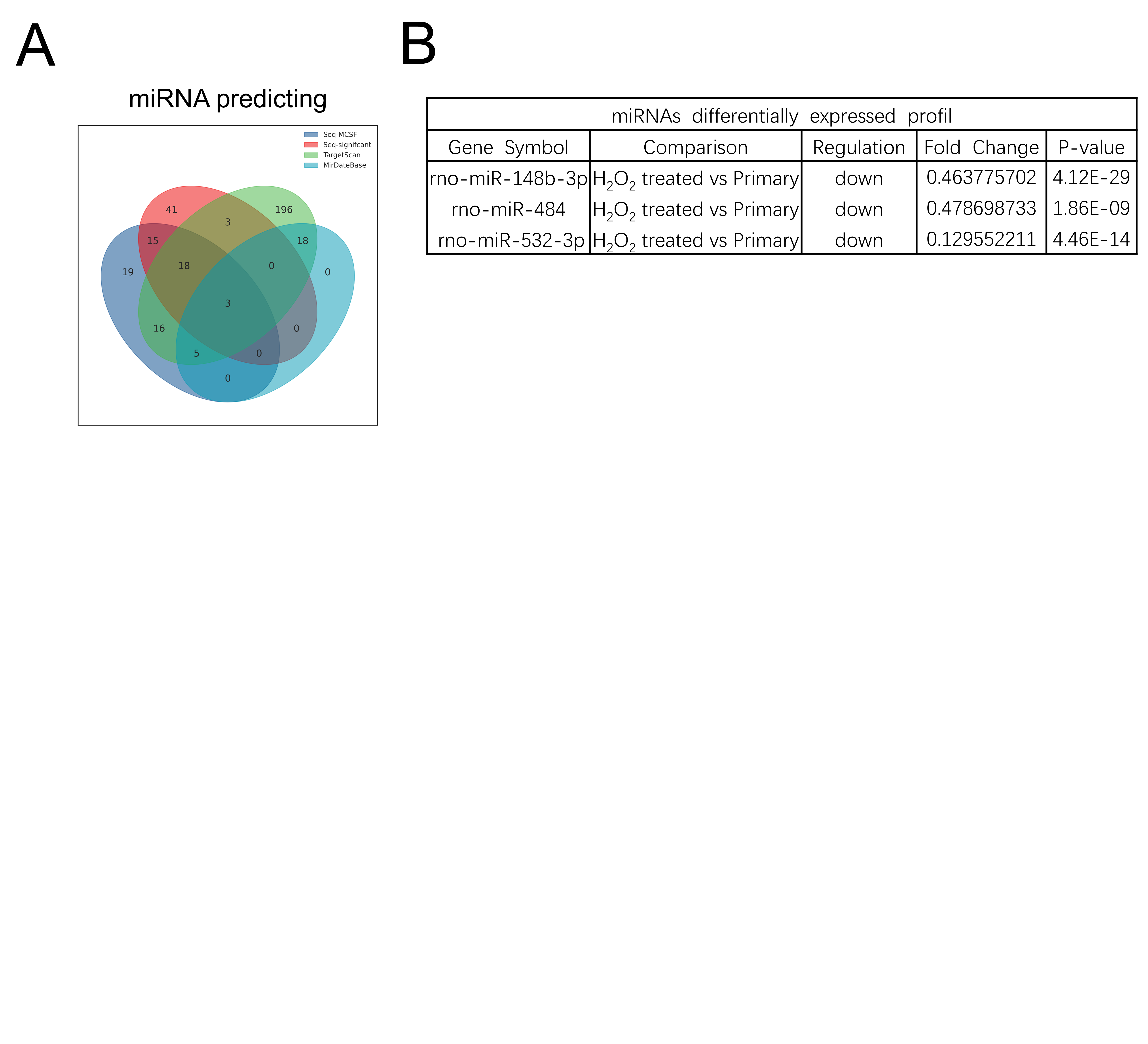


**Supplementary Figure 7. Prediction of upstream miRNAs.** (A) Bioinformatic analysis was used to predict miRNAs. (B) miRNAs differentially expressed profile.


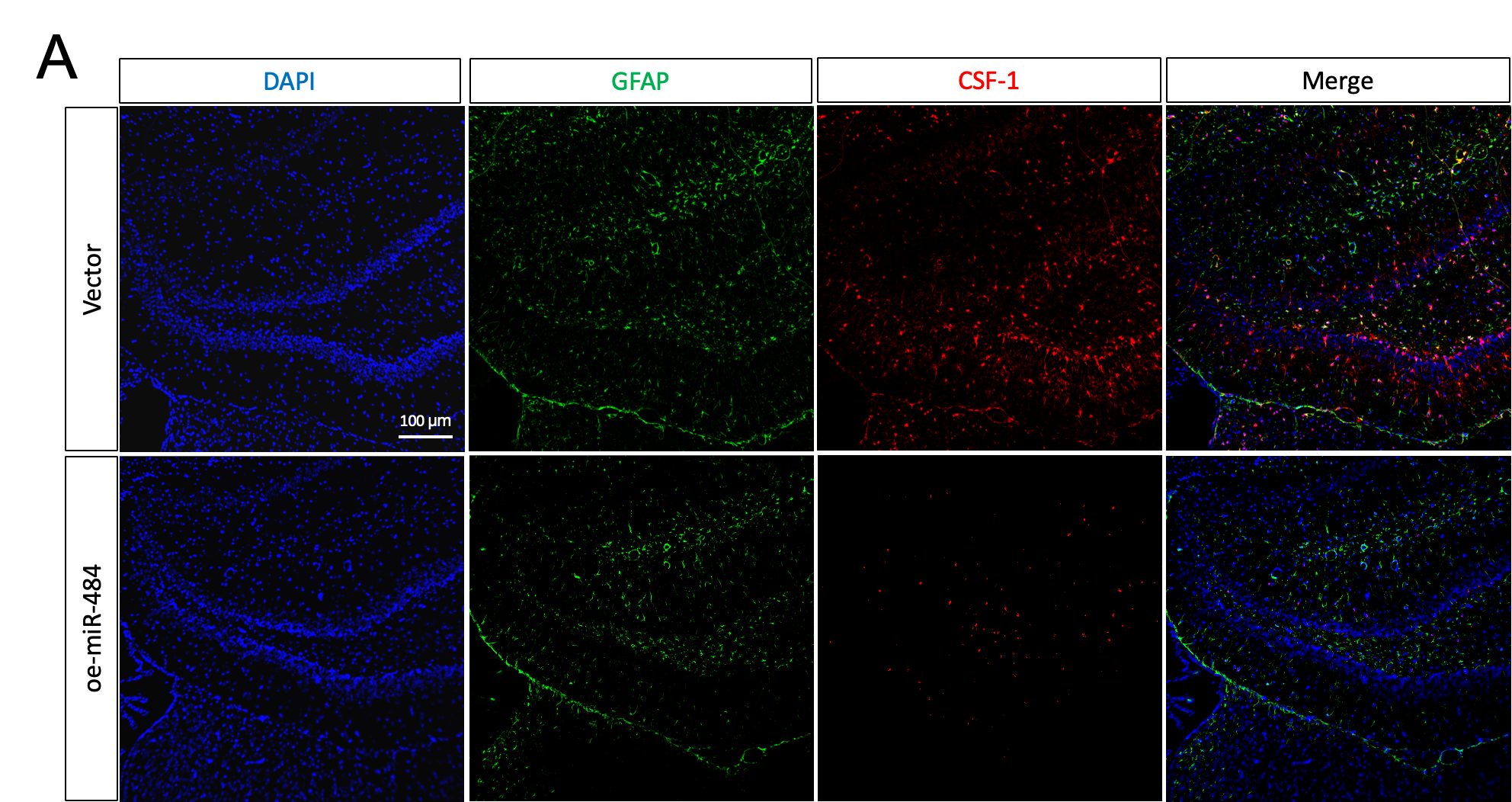


**Supplementary Figure 8. The CSF-1 expression in AAV2/9-GFAP-miR-484 transfected rat brains.** (A) Immunofluorescence images of CSF-1 expression in AAV2/9-GFAP-miR-484 transfected brains.

**Supplementary Tables. 1 Reagents used in this study**

| **Reagents** | **Company** | **Cat No.** | **application** |
| --- | --- | --- | --- |
| Mouse anti-Nestin | Abcam | ab6142 | Immunofluorescence staining |
| Rabbit anti-Caspase-3 | Thermo Fisher | PA5-77887 | Immunofluorescence staining |
| Rabbit anti-Ki67 | Abcam | ab16667 | Immunofluorescence staining |
| Rabbit anti-CSF-1 | Abnova | PAB16618 | Immunofluorescence staining |
| Rat anti-GFAP | Abcam | ab279291 | Immunofluorescence staining |
| Rabbit anti-beta III Tubulin | Abcam | ab18207 | Immunofluorescence staining |
| DAPI | Thermo Fisher | D1306 | Immunofluorescence staining |
| Rabbit anti-Phospho-Histone H2A.X | Cell Signaling Technology | 9718S | Western blot |
| Rabbit anti-p21 | Abcam | ab109199 | Western blot |
| Rabbit anti-p53 | Abcam | ab131442 | Western blot |
| Rabbit anti-CSF-1 | Abnova | PAB16618 | Western blot |
| AF 488 donkey anti-Mouse IgG | Abcam | ab150113 | Secondary Antibody |
| AF 488 donkey anti-Rabbit IgG | Abcam | ab150077 | Secondary Antibody |
| AF 594 donkey anti-Mouse IgG | Abcam | ab150116 | Secondary Antibody |
| AF 594 donkey anti-Rabbit IgG | Abcam | ab150080 | Secondary Antibody |
| HRP-linked Anti-Mouse | Cell Signaling Technology | 7076 | Secondary Antibody |
| HRP-linked Anti-Rabbit | Cell Signaling Technology | 7074 | Secondary Antibody |
| Dulbecco’s Modified Eagle Medium/F12 (DMEM/F12) | Gibco, USA | 11320-033 | cell culture |
| Fetal Bovine Serum (FBS) | Gibco, USA | 10100-147 | cell culture |
| B-27 Serum-Free Supplement (50×) | Gibco, USA | 17504-044 | cell culture |
| Epidermal growth factor (EGF) | Sigma-Aldrich, USA | E5160-100UG | cell culture |
| Basic fibroblast growth factor (bFGF) | Gibco, USA | 400-29-1MG | cell culture |
| Penicillin/Streptomycin | Gibco, USA | 15070-063 | cell culture |
| Poly-L-Lysine (PLL) | Sigma-Aldrich, USA | P4707 | cell culture |
| CSF-1 recombinant protein | Gibco, USA | 400-28-50UG | cell culture |
| 3%H_2_O_2_ | Sigma-Aldrich, USA | 7722-84-1 | senescence induction |
| CSF-1 | MULTI SCIENCES | EK2144 | Elisa |
| Senescence β-Galactosidase Staining Kit | Beyotime | C0602 | senescence staining |

**Supplementary Table. 2 PCR sequences.**

| **Gene** | **Forward primer (5′-3′)** | **Reverse primer(5′-3′)** |
| --- | --- | --- |
| *Csf-1* | TGGACGATCCCGTTTGCTAC | ACACAGGCCTCGTTCTGTTC |
| *Ki67* | CCATTAACAAGAGTGAGGGAGTG | TGAGTGGAGTATTAGGAGGCAAG |
| *Caspase-3* | TGGCACACGGGACTTGGAAAG | CCAGCGATGACTCAGCACCTC |
| *Tuj1* | TCCGCCTGCCTCTTCGTCTC | GGTCTATGCCATGCTCGTCACTG |
| *p16* | CCAGCACACGGAGTTCCATCAC | GAGCATGAGCAGGAAGCCATAGC |
| *p21* | TCCTCTCAACGCCATTTATGCCAAG | ACCTTCTCACACAGCCTCAGTCC |
| *Sox2* | TAGAGCTAGACTCCGGGCGATGA | TTGCCTTAAACAAGACCACGAAA |
| *Nestin* | AGGAAGAAGCTGCAGCAGAG | TTCACCTGCTTGGGCTCTAT |
| *Il-1β* | TGTCTGACCCATGTGAGCTG | TTTGGGATCCACACTCTCCA |
| *Il-6* | CAGAGGATACCACCCACAACAGA | CAGTGCATCATCGCTGTTCATACA |
| *Il-4* | CGTGATGTACCTCCGTGCTT | GTGAGTTCAGACCGCTGACA |
| *Il-10* | ACTGCTATGTTGCCTGCTCTT | ATGTGGGTCTGGCTGACTGG |
| *rno-miR-148b-3p* | ATGTGCGTCAGTGCATCACAGA | ATCCAGTGCAGGGTCCGAGG |
| *rno-miR-532-3p* | ACCATTCACCTCCCACACCCA | ATCCAGTGCAGGGTCCGAGG |
| *rno-miR-484* | AGTATCCTCAGGCTCAGTCCCC | ATCCAGTGCAGGGTCCGAGG |
| *U6* | CATGTACGTTGCTATCCAGGC | CTCCTTAATGTCACGCACGAT |
| *Gapdh* | GCAAGTTCAACGGCACAG | GCCAGTAGACTCCACGACAT |
